# Major cardiovascular events after bone marrow mononuclear cell transplantation following acute myocardial infarction: an updated post-BAMI meta-analysis of randomized controlled trials

**DOI:** 10.1186/s12872-022-02701-x

**Published:** 2022-06-09

**Authors:** Armin Attar, Alireza Hosseinpour, Hamidreza Hosseinpour, Asma Kazemi

**Affiliations:** 1 Department of Cardiovascular Medicine, School of Medicine, Shiraz University of Medical Sciences, Shiraz, Iran; 2 Faculty of Medicine, Shiraz University of Medical Sciences, Shiraz, Iran; 3 Nutrition Research Center, Shiraz University of Medical Sciences, Shiraz, Iran

**Keywords:** Stem cell, Myocardial infarction, Heart failure, Bone marrow mononuclear cell

## Abstract

**Background:**

The effect of bone marrow-derived mononuclear cells (BM-MNCs) after acute myocardial infarction (AMI) on myocardial function indices such as left ventricular ejection fraction has been widely studied. However, the effect of this intervention on major adverse cardiovascular events (MACE) was not the principal purpose of most investigations and its role is unclear. The aim of this study was to investigate the possible long-term clinical efficacy of BM-MNCs on MACE after AMI.

**Methods:**

A comprehensive search was conducted through electronic databases for potentially eligible randomized trials investigating the impact of BM-MNC therapy following acute MI on clinical outcomes. Risk of bias of the eligible studies was assessed using the Cochrane Collaboration’s tool. The effect of treatment was displayed by risk ratio (RR) and its 95% confidence interval (CI) using random-effects model.

**Results:**

Initial database searching found 1540 records and 23 clinical trials with a total of 2286 participants eligible for meta-analysis. Injection of BM-MNCs was associated with lower risk of composite end points of hospitalization for congestive heart failure (CHF), re-infarction, and cardiac-related mortality (91/1191 vs. 111/812, RR = 0.643, 95% CI = 0.489 to 0.845, *p* = 0.002). This effect was derived from both reduction of CHF (47/1220 vs. 62/841, RR = 0.568, 95% CI = 0.382 to 0.844, *p* = 0.005) and re-infarction rate (23/1159 vs. 30/775, RR = 0.583, 95% CI = 0.343 to 0.991, *p* = 0.046), but not cardiac-related mortality (28/1290 vs. 31/871, RR = 0.722, 95% CI = 0.436 to 1.197, *p* = 0.207).

**Conclusion:**

This is the first meta-analysis focused on the cardiovascular outcomes of stem cell therapy after AMI and it revealed that transplantation of BM-MNCs may reduce composite endpoint of hospitalization for CHF, re-infarction, and cardiac related mortality driven mainly by reducing reinfarction and hospitalization for heart failure rates but not cardiovascular mortality.

**Supplementary Information:**

The online version contains supplementary material available at 10.1186/s12872-022-02701-x.

## Background

Myocardial infarction (MI) represents the leading cause of mortality worldwide [1]. With a reduction in the rate of mortality due to MIs in recent decades, the incidence of heart failure (HF) has been on the rise [2]. This incidence ranges between 14 and 36% among those hospitalized due to an acute MI (AMI) [3]. HF exerts a considerable effect on healthcare systems in America, accounting for 6 million cases, 300,000 deaths, and roughly 40 billion USD worth of costs every year [4]. Despite the therapeutic efforts [5], post-MI HF still leads to a high rate of morbidity and mortality [6, 7]. Although we have been successful in prolonging the life of HF patients and relieving symptoms, we are yet to regenerate the infarcted cardiac tissues. Hence, a gap exists in the literature as restoring the standard histological architecture of the heart should theoretically lead to improved outcomes for patients with MI-induced HF [6]. This may be possible using stem cell-based therapies [8].

For over two decades, autologous cell-based treatments using bone marrow mononuclear cells (BM-MNC) have been assessed in managing cardiovascular diseases through preclinical and clinical studies. However, phase III trials have been infrequent and most of them have only assessed paraclinical outcomes such as left ventricular ejection fraction (LVEF); also, the infarct size and trials with clinical endpoints are rare.

The BAMI trial was the first phase III trial conducted to clarify whether or not post-MI intracoronary transplantation of BM-MNCs would reduce all-cause mortality [9]. All-cause mortality after two years was 3.26% [n = 6; 95% confidence interval (CI) 1.48–7.12%] with BM-MNCs compared to 3.82% (n = 7; 95% CI 1.84–7.84%) with optimal medical therapy. Importantly, the investigators noticed that only five patients (2.7%, 95% CI 1.0–5.9%) who received BM-MNCs were hospitalized due to HF during the two years of follow-up compared with 15 patients (8.1%, CI 4.7–12.5%) who received optimal medical therapy (HR: 0.33, 95% CI: 0.12–0.88), representing the sole clinical benefit observed. Since the effect of BM-MNC transplantation after AMI on major cardiovascular outcomes is poorly studied and to the best of our knowledge no meta-analysis has focused specifically on this issue, in this meta-analysis, we have investigated the clinical outcomes of patients who had undergone autologous BM-MNC transplantation after AMI.

## Methods

This meta-analysis was registered in PROSPERO (CRD42022295741) and it was prepared and reported using the recommendations made by the Preferred Reporting Items for Systematic reviews and Meta-Analyses (PRISMA) statement [10].

### Eligibility criteria

Potential eligible studies were all the randomized controlled trials which performed autologous transplantation of BM-MNCs following a successful coronary angioplasty using stent implantation in patients diagnosed with acute ST-segment elevation MI. Patients undergoing stem cell therapy who were compared with a control group of acute MI patients receiving standard therapy with or without intracoronary injection of placebo were considered for inclusion. Studies were excluded if they did not include a control arm, were not randomized, had less than 6 months of follow-up, used any other stem cells than bone-marrow mononuclear cells as the stem cell therapy, and did not compare long term adverse clinical events including hospitalization due to CHF, recurrent MI, and composite endpoints (cardiac death, CHF, and MI) between the intervention and control groups. The primary outcomes were major adverse cardiovascular events (MACE) including rehospitalization for CHF, recurrence of MI, cardiac-related death, and the composite endpoints separately. The clinical outcomes were assessed at the longest available follow up (at least 6 months). Comparisons of the left ventricular function indices including LVEF, left ventricular end-diastolic volume (LVEDV), and left ventricular end-systolic volume (LVESV) between 3 and 12 months after stem cell therapy in the intervention group and the control arm were listed as secondary outcomes of interest.

### Search strategy for identification of eligible studies

We conducted a comprehensive search through PubMed, Embase, and Cochrane Central Register of Controlled Trials which were last performed on 5 April 2021 by using one or a combination of keywords including “myocardial infarction”, “coronary artery disease”, “stem cell”, “mononuclear cell”, “bone marrow”, and “heart failure”. Articles in English with no further restriction in sample size or time frame were screened for eligibility. Bibliographies were screened to find any other relevant studies. All the abstracts and titles of the identified studies were screened by two independent reviewers (AA and AH), and the full texts of the possible eligible ones were considered suitable for meta-analysis if they met the inclusion criteria. In any case of discrepancy, disagreements were resolved by discussion with a third investigator (HH).

### Data extraction

A reviewer (AH) independently collected the study information including trial characteristics (authors, trial name, and year of publication), sample size of the intervention and control groups, information regarding the features of intervention (stem cell injection time, dose of injection, route of injection), primary outcomes of both control and intervention arms (rehospitalization due to CHF, reinfarction, cardiac-related mortality, and composite of hospitalization, MI and cardiac death either stated in the study or calculated by the reviewer), and characteristics of secondary outcomes (LVEF, LVEDV, LVESV, change in the mentioned markers over the follow up period, and the modality used for measurement of left ventricular (LV) indices. If the change in the mentioned markers was measured for multiple times over the follow up period, values at 6 months of follow up were extracted for analyses. Then, a second investigator (AA) evaluated the accuracy and consistency of the extracted data. Disagreements were solved by discussion between the authors.

### Risk of bias and quality appraisal

The quality of the selected studies was assessed by two authors (AA and AH) independently, using the Cochrane Collaboration’s tool for assessing risk of bias in randomized trials [11]. We evaluated the studies for selection, performance, detection, attrition, and reporting bias with Review Manager (RevMan 5.1.7) Software and rated the status of bias as low, unclear, or high risk. If there were any disagreements, the authors resolved them by discussion.

### Statistical analysis

The extracted data from the enrolled eligible studies were entered to a pre-designed Microsoft Excel spreadsheet and all the analyses were conducted using Stata software version 13 (StataCorp LP, College Station, TX, USA). For the primary endpoints (hospitalization for CHF, myocardial reinfarction, cardiac-related mortality, and composite endpoints), we reported the risk ratio (RR) and its 95% confidence interval (CI) as the treatment effect. Also, subgroup analyses for the primary endpoints were made according to the time of stem cell injection (*early* group defined as patients receiving stem cell < 11 days and *late* group ≥ 11 days) and dosage of therapy (≥ 10^8^: *high* dose and < 10^8^: *low* dose) to study the effect of time and dose. Moreover, we expressed continuous data for secondary endpoints as weighted mean difference (WMD) and 95% CI. The publication bias was assessed using funnel plots and Egger’s test. The I^2^ values were calculated for measuring the amount of heterogeneity. Random-effects model was used for all the analyses. The studies were different regarding the time and dosing of BM-MNCs transplantation. Also, some studies divided the intervention group into subgroups with different times of injection (early or late) and injection dose (high dose and low dose). Since timing and dosage of stem cell therapy could impact the clinical outcomes of the participants, we grouped the studies according to the time of injection (≥ 11 days after revascularization as the *late* group or < 11 days defined as the *early* group) and dosage of stem cell therapy (*high dose* group was defined as a median or mean of ≥ 10^8^ BM-MNC injected and lower number of cells was considered as *low dose* group) and analyzed the primary outcomes of interest between the subgroups. Furthermore, different modalities including echocardiography, LV angiography, cardiac magnetic resonance (CMR) imaging, and Single-photon emission computed tomography (SPECT) were used for measurement of LV indices among the studies. Thus, in addition to the main analysis, we compared the LV indices in subgroups for each modality. Random-effects meta-regression was performed to explore the potential linear associations between baseline characteristics (age and gender) and primary outcomes of interest.

## Results

### Literature search and study characteristics

**Fig. 1:**
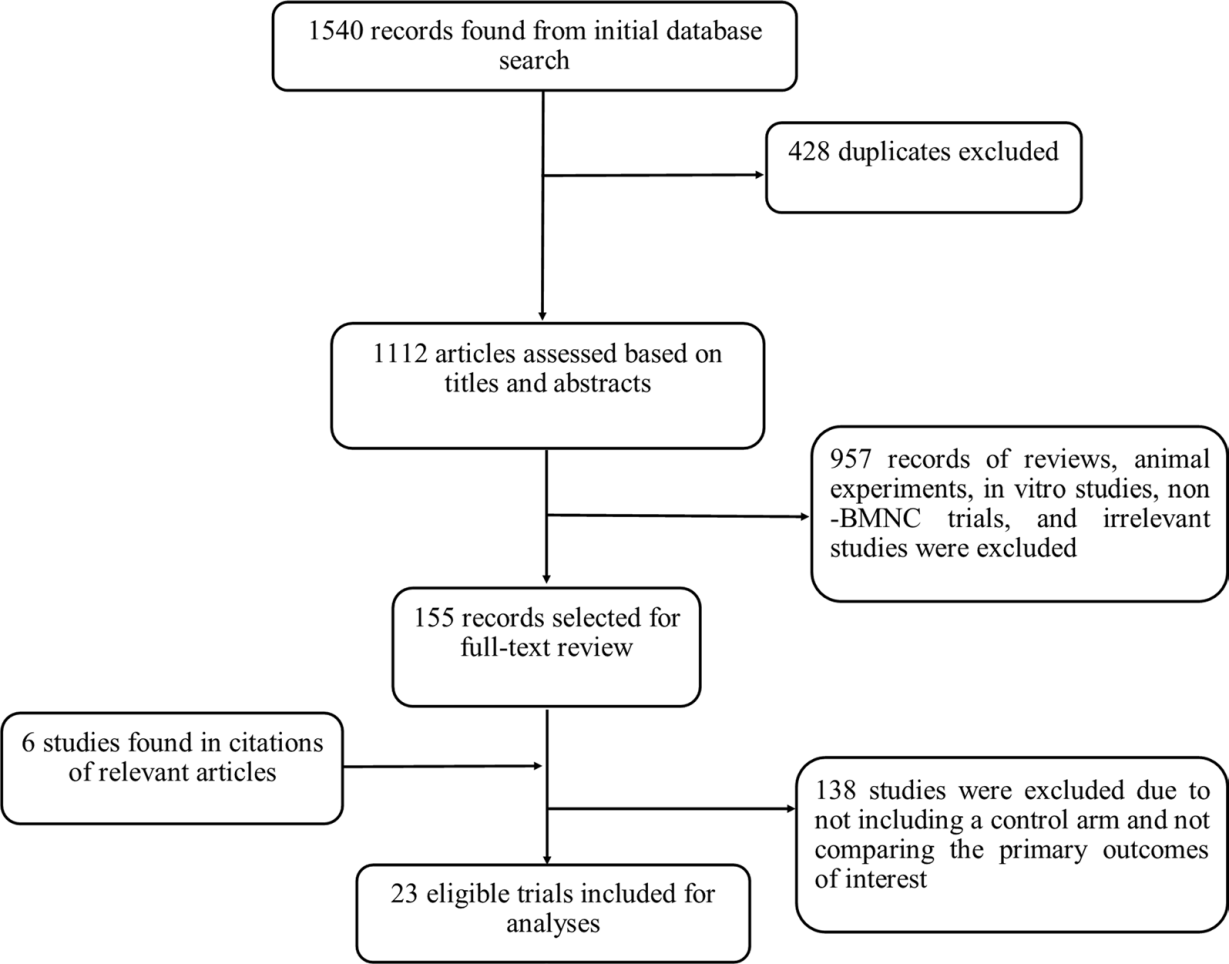
Flow diagram of the eligible studies included in the meta-analysis

We identified a total of 1540 records through electronic searches of PubMed, Embase, and Cochrane database. After removal of the duplicates, titles and abstracts of 1112 records were screened for potential eligibility. At this stage, 957 records were excluded (letters, reviews, in vitro and animal studies, and irrelevant topics), and full texts of 155 articles were selected for screening. After detailed assessment of the potentially eligible studies, 23 trials met all the inclusion criteria and were considered eligible for the meta-analyses (Fig. 1) [9, 12–33]. The eligible studies included a total of 2286 participants (1402 receiving BM-MNC therapy and 884 in the placebo group). The most frequent comorbidities of the participants included hypertension, hyperlipidemia, and diabetes. No inclusion or exclusion criteria was set for pre-existing conditions of the patients. The injection time of BM-MNCs ranged from 24 h to 3 months after AMI. All the trials measured the primary outcomes of interest of this review (CHF needing hospitalization, reinfarction, and mortality), and their follow up period ranged from 6 to 60 months. For the secondary outcomes (LV function indices), the follow ups ranged from 3 to 12 months. Six studies divided the patients receiving BM-MNCs into different subgroups: one trial giving BM-MNCs based on normoxia or hypoxia-preconditioning of the stem cells [14], two studies based on the time of injection [15, 22], one based on the dosage (low dose and high dose group) [18], one according to the type of mononuclear cell [29], and one based on the dosage and the status of radiation given to the cells [30]. In all the included trials, the route of injection was intracoronary. Characteristics of the trials are summarized in Table 1.

**Table 1:** Characteristics of the included studies

Study	Trial Name	Country	Sample size		Mean age		Male (%)		Baseline LVEF		Injection time interval(d)	Modality	F/U for clinical events(m)
			BM-MNC	Control	BM-MNC	Control	BM-MNC	Control	BM-MNC	Control			
Meyer et al. [27]	BOOST	Germany	30	30	53.4 ± 14.8	59.2 ± 13.5	67	73	50 ± 10	51.3 ± 9.3	4.8 ± 1.3	CMR	61
Assmus et al. [12]	REAPIR-AMI	Germany	101	103	55 ± 11	57 ± 11	82	82	47.5 ± 10	46.7 ± 10.3	4.4 ± 1.3	LV angiography	60
Beitnes et al. [32]	ASTAMI	Norway	50	50	58.1 ± 8.5	56.7 ± 9.6	84	84	45.7 ± 9.4	46.9 ± 9.6	4–8	Echo/CMR	36
Benedek et al. [33]	-	Romania	9	9	53.55 ± 15.08	61 ± 10.06	77.77	55.55	41.66 ± 3.5	39.7 ± 3	21–90	Echo	48
Delewi et al. [13]	HEBE	Netherlands	69	65	56 ± 9	55 ± 10	84	86	43.7 ± 9	42.4 ± 8.3	8	CMR	60
Hu et al. [14]	CHINA-AMI	China	22	14	60.45 ± 11.4	60.62 ± 10.85	86.5	64	53.8 ± 11.5	57.1 ± 11.6	5	Echo/SPECT	12
Huang et al. [15]	-	China	79	25	58.55 ± 8.72	58.8 ± 8.4	91	88	43.65 ± 5.21	43.5 ± 3.5	1–30	Echo/SPECT	12
Huikuri et al. [16]	FINCELL	Finland	40	40	60 ± 10	59 ± 10	90	85	59 ± 11	62 ± 12	2–6	LV angiography/echo	6
Lamriault et al. [17]	BONAMI	France	59	42	56 ± 12	55 ± 11	80.8	89.8	38.1 ± 7.9	39.8 ± 7	9.3 ± 1.7	Echo	12
Mathur et al. [9]	BAMI	UK	185	190	59 ± 11	60 ± 11	83.78	77.37	39 ± 5	39 ± 5	2–8	Echo	24
Meluzin et al. [18]	-	Czech	40	20	54 ± 2	55 ± 2	92.5	90	40.5 ± 8.94	40 ± 8.94	3–8	Echo/SPECT	12
Plewka et al. [19]	-	Poland	40	20	56 ± 9	56 ± 9	67	75	35 ± 6	33 ± 7	7	Echo	24
San Roman et al. [20]	TECAM	Spain	30	31	54 ± 11	57 ± 11	97	90	49 ± 8	47 ± 8	3–5	CMR/LV angiography	12
Skalicka et al. [21]	-	Czech	17	10	61 ± 14	54 ± 10	71	100	39.2 ± 9.2	39.4 ± 5.6	4–11	Echo	24
Sürder et al. [22]	-	Switzerland	133	67	58.53 ± 14.77	56 ± 14.5	84	83.6	36.4 ± 8.9	40 ± 9.9	5–28	CMR	12
Traverse et al. [23]	TIME (phase I)	USA	30	10	52.5 ± 15.56	57.5 ± 3.7	83	60	49 ± 9.5	48.6 ± 8.5	3–10	CMR/Echo	6
Traverse et al. [24]	LateTIME	USA	58	29	57.6 ± 11	54.6 ± 11	79	90	48.7 ± 12	45.3 ± 9.9	14–21	CMR	6
Traverse et al. [25]	TIME	USA	58	27	55.9 ± 11	56.4 ± 10.4	88	86	45.9 ± 9.4	46.9 ± 8.7	3–7	CMR	24
Wöhrle et al. [26]	-	Germany	29	13	61 ± 8.1	61.1 ± 9.3	90	62	53.5 ± 9.3	55.7 ± 9.4	5–7	CMR	6
Piepoli et al. [28]	Cardiac study	Italy	19	19	63.1 ± 2.4	67 ± 2.7	68.4	68.4	38.9 ± 1.3	38.4 ± 1.5	4–7	Echo/SPECT	12
Tendera et al. [29]	REGENT	Poland	160	40	56.5 ± 29.98	59 ± 26.67	67	75	36 ± 21.2	39 ± 15.56	3–12	CMR	6
Wollert et al. [30]	BOOST-2	Germany	127	26	55.46 ± 9.83	55 ± 9	85	92	44.3 ± 8.48	47.8 ± 6.7	7.1 ± 2.6	CMR	6
Yao et al. [31]	-	China	27	12	51.7 ± 6.4	52.7 ± 7.8	81	92	33.2 ± 3.9	32.3 ± 2	3d-3 m	CMR	12

### Risk of bias in individual studies

The quality assessment of the enrolled studies was performed, as illustrated in Additional file 1: Fig. S1. Sixteen trials [9, 12–14, 16, 17, 20, 22–25, 27, 28, 30–32] reported their method for random sequence generation and seven trials [15, 18, 19, 21, 26, 29, 33] did not mention a clear method of randomization. Also, eleven trials were at low risk for proper statement of allocation concealment [9, 12, 14–16, 18, 23–25, 31, 32]. Eight trials did not perform blinding of either participants or personnel [9, 13, 15, 17, 20–22, 29] and seven were unknown regarding the blinding process [18, 19, 27, 28, 31–33]. Masking was not done or was unclear for outcome assessors of four trials [15, 19, 29, 33]. Six studies [15, 19–22, 30] were high risk for attrition bias and two trials [18, 28] were at unclear risk. Out of all the included trials, only three [18, 26, 31] were unclear regarding selective reporting of outcomes. Also, we evaluated the possibility of bias by assessing funnel plots and Egger’s test. For the primary outcomes of interest (rehospitalization for CHF and composite endpoints), *p-value* did not reach a significant level (*p* = 0.082 and *p* = 0.120, respectively), and funnel plots showed symmetrical distribution (Additional file 1: Figs. S2, S3).

### Hospitalization for heart failure

**Fig. 2:**
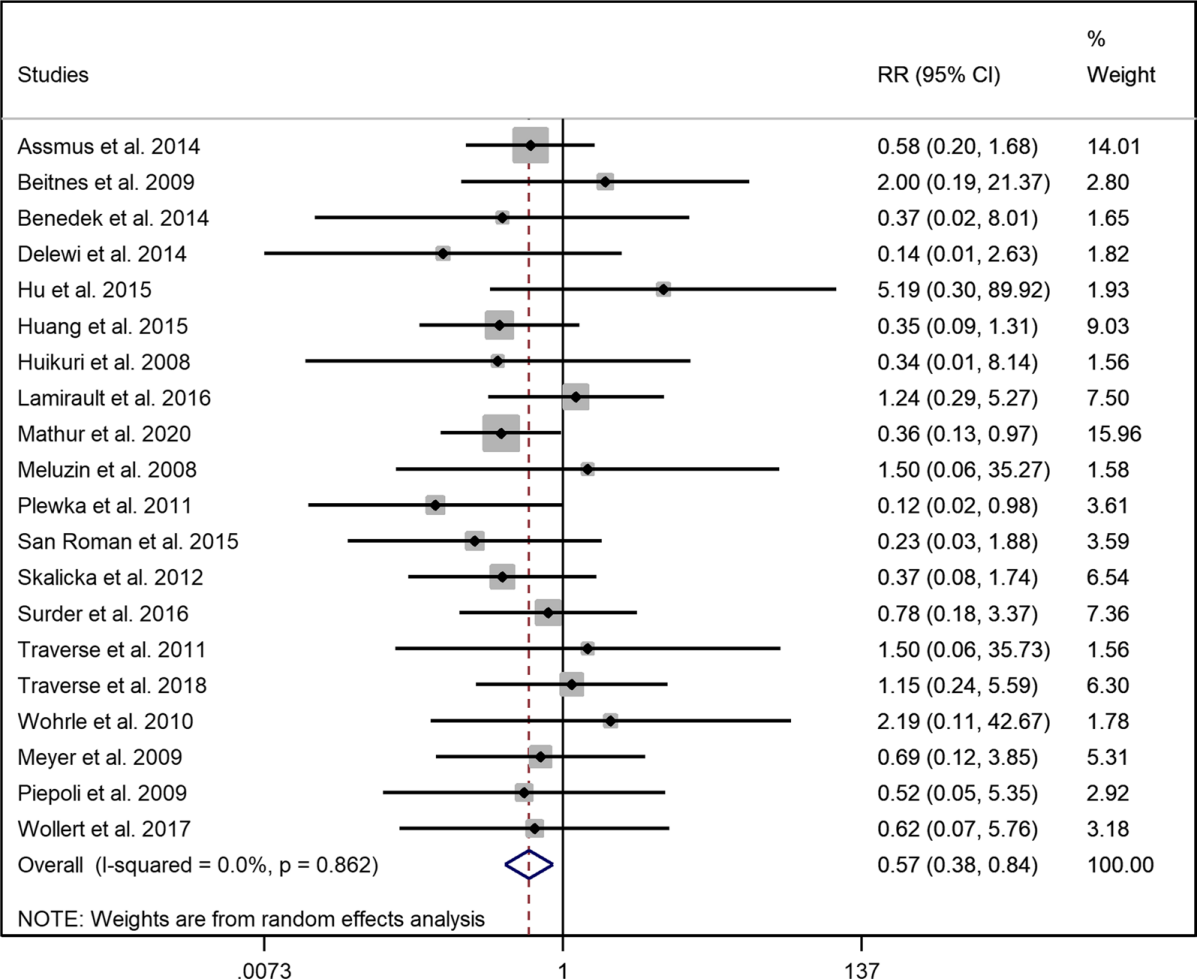
Forest plot demonstrating relative risk of hospitalization for CHF compared between the intervention and control groups (RR: Risk ratio)

Twenty trials reported the number of cases needing rehospitalization due to CHF in both intervention and placebo groups during their follow-up (Duration of follow-up period ranged from 6 to 61 months). Overall, there was a significantly lower risk of hospitalization for CHF in the intervention group compared to the control group which received placebo (RR = 0.568, 95% CI = 0.382 to 0.844, *p* = 0.005, I^2^ = 0.00%) (Fig. 2). Subgroup analysis showed that early injection of BM-MNCs could lower the risk of hospitalization (RR 0.539, 95% CI = 0.354 to 0.819, *p* = 0.004, I^2^ = 0.00%), whereas there was no significant difference of hospitalization compared to the control group in the intervention group with late injection of BM-MNCs (RR = 0.810, 95% CI = 0.298 to 2.198, *p* = 0.678, I^2^ = 0.00%) (Additional file 1: Fig. S4). Also, there was no evidence for a difference in hospitalization in the low dose group (RR = 0.998, 95% CI = 0.364 to 2.735, *p* = 0.997, I^2^ = 0.00%) contrary to high dose group; the risk of hospitalization was significantly lower (RR = 0.518, 95% CI = 0.337 to 0.798, *p* = 0.003, I^2^ = 0.00%) (Additional file 1: Fig. S5).

### Myocardial reinfarction

**Fig. 3:**
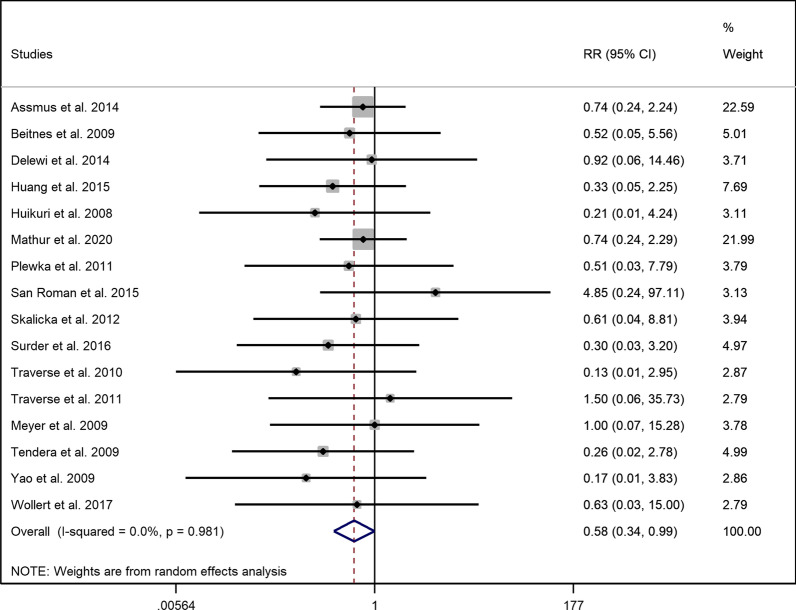
Forest plot demonstrating relative risk of myocardial reinfarction between the intervention and control group (RR: Risk ratio)

Incidence of myocardial reinfarction was reported in eighteen enrolled studies. Two trials stated no recurrence of MI in their study [14, 33]. Similar to hospitalization for CHF, there was a significant difference regarding the occurrence of reinfarction between the intervention and placebo group (RR = 0.583, 95% CI = 0.343 to 0.991 *p* = 0.046) with no evidence of heterogeneity (I^2^ = 0.00%) (Fig. 3). Subgroup analysis of early and late injection of the stem cells revealed that both results of early and late injection were not different compared to the control group (*early*: RR = 0.585, 95% CI = 0.339 to 1.008, *p* = 0.054, I^2^ = 0.00% and *late*: RR = 0.555, 95% CI = 0.113 to 2.741, *p* = 0.470, I^2^ = 0.00% (Additional file 1: Fig. S6)). Moreover, there was evidence for a difference in the risk of MI in the group with high dose of injection in contrast to the group with low dose of injection (*High dose*: RR = 0.566, 95% CI = 0.326 to 0.984, *p* = 0.044, I^2^ = 0.00% and *low dose*: RR = 1.309, 95% CI = 0.149 to 11.490, *p* = 0.808, I^2^ = 25.00% (Additional file 1: Fig. S7)).

### Cardiac-related mortality

**Fig. 4:**
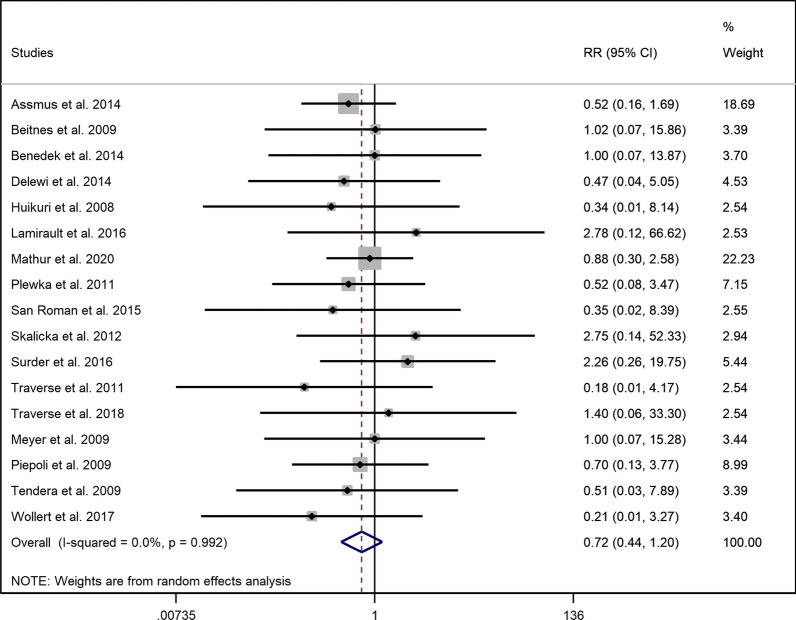
Forest plot demonstrating relative risk of cardiac-related mortality between the intervention and control group (RR: Risk ratio)

Twenty-one studies stated the number of cardiac-related mortality in their trials although in some studies the incidence of cardiac-related and all-cause mortality was not differentiated. The incidence of cardiac death appeared to be not significantly different between the two study groups (RR = 0.722, 95% CI = 0.436 to 1.197, *p* = 0.207, I^2^ = 0.00%) (Fig. 4). Subgroup analysis of cardiac death in both early and late injection also remained insignificant with no evidence of heterogeneity (I^2^ = 0.00%) (*Early*: RR = 0.750, 95% CI = 0.444 to 1.265, *p* = 0.280 and *late*: RR = 0.693, 95% CI = 0.136 to 3.533, *p* = 0.659 (Additional file 1: Fig. S8)). Similarly, no difference was found regarding the risk of cardiac death in the high and low dose group compared to controls (*High dose:* RR = 0.701, 95% CI = 0.413 to 1.189, *p* = 0.187 and *low dose*: RR = 1.001, 95% CI = 0.176 to 5.679, *p* = 0.999 (Additional file 1: Fig. S9) (I^2^ = 0.00% in both analyses)).

### Composite endpoints (hospitalization for heart failure, myocardial reinfarction, and cardiac-related mortality)

**Fig. 5:**
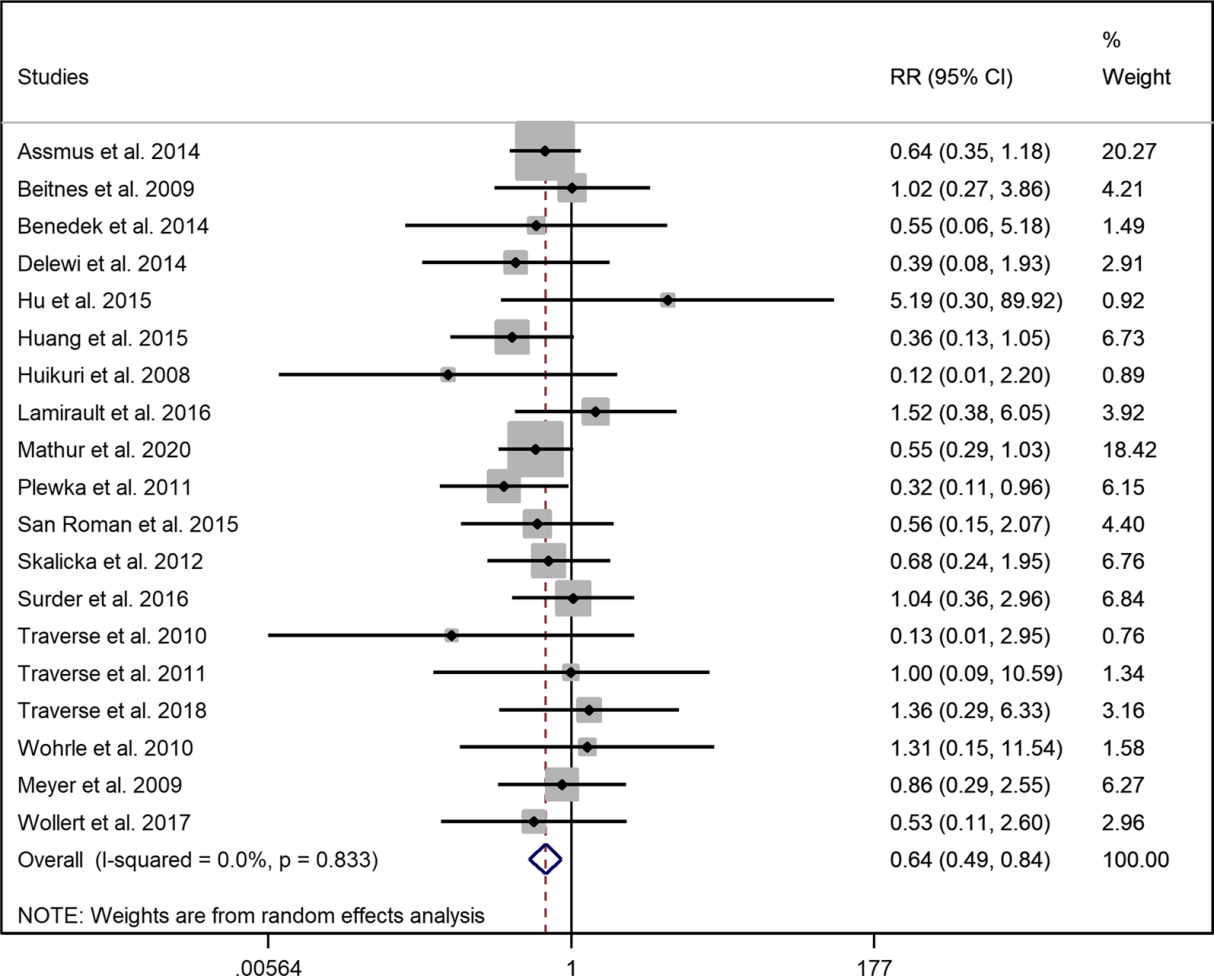
Forest plot demonstrating relative risk of composite endpoints between intervention and control group (RR: Risk ratio)

As defined before, the composite endpoints could be calculated in nineteen trials. There was evidence that autologous injection of BM-MNCs could lower the risk of composite endpoints in the intervention group when compared to the control arm (RR = 0.643, 95% CI = 0.489 to 0.845, *p* = 0.002), and there was no evidence of heterogeneity (I^2^ = 0.00%) (Fig. 5). Patients who had received BM-MNCs earlier than 11 days (early group) had a lower risk of composite endpoint (RR = 0.636, 95% CI = 0.479 to 0.845, *p* = 0.002, I^2^ = 0.00%), whereas there was no evidence of lower risk of composite endpoints in the late group who received stem cell therapy compared to standard therapy (RR = 0.748, 95% CI = 0.340 to 1.644, *p* = 0.470, I^2^ = 0.00%) (Additional file 1: Fig. S10). Furthermore, patients who received high doses of mononuclear cells were at lower risks of composite end points (RR = 0.609, 95% CI = 0.455 to 0.816, *p* = 0.001) although those in the low dose group were not significantly different compared to the controls (RR = 0.936, 95% CI = 0.432 to 2.028, *p* = 0.868) (Additional file 1: Fig. S11), both with no evidence of heterogeneity (I^2^ = 0.00%).

### Left ventricular ejection fraction

Twenty-one studies measured the change in LVEF in the follow-up period (ranging from 3 to 12 months). LVEF improved significantly in patients in the intervention group compared to the control arm (WMD = 1.695%, 95% CI = 0.681 to 2.710, *p* = 0.001) and high level of heterogeneity (I^2^ = 65.1%) (Additional file 1: Fig. S12). Publication bias was not significant according to both Begg’s test (*p* = 0.880) and Egger’s test (*p* = 0.208).

#### Echocardiography

Ten trials used echocardiography for measuring LVEF [14–17, 19, 21, 23, 28, 32, 33] and three of them measured the change of LVEF from baseline in the follow up [14, 15, 34] (We used the data from a serial publication for one of the trials (Beitnes et al. [32]) for change of LVEF). An improvement in LVEF associated with cell therapy was found (WMD = 1.550%, 95% CI = 0.408 to 2.692, *p* = 0.008, I^2^ = 37.6%).

#### CMR

CMR imaging was used for measurement of LV markers in 13 studies [13, 20, 22–26, 29–32]. After exclusion of the studies with low correlation with others, pooled analysis showed no significant difference in LVEF associated with stem cell therapy (WMD = 0.981%, 95% CI = -0.966 to 2.929, *p* = 0.323, I^2^ = 76.3%).

#### LV angiography

Analysis of change in the LVEF of the studies using LV angiography [12, 16, 20] showed evidence of improvement in LVEF linked with BM-MNCs injection (WMD = 3.192%, 95% CI = 0.509 to 5.874, *p* = 0.020, I^2^ = 23.0%).

#### SPECT

Two studies used SPECT for measuring LVEF values in baseline and follow-ups [18, 28], and the observed change was found to be not significant (WMD = 3.036%, 95% CI = − 1.285 to 7.357, *p* = 0.168, I^2^ = 18.6%) (Additional file 1: Fig. S13).

#### Other echocardiographic parameters

There was not a significant correlation between stem cell therapy and improvement in LVEDV (WMD = -2.940, 95% CI = − 6.505 to 0.625, *p* = 0.106, I^2^ = 54.1%) (Additional file 1: Figs. S14, S15) and low possibility of publication bias according to Egger’s test (*p* = 0.211). Moreover, there was an association between stem cell therapy and changes in LVESV (WMD = − 2.376, 95% CI = − 3.534 to − 1.218, p < 0.001, I^2^ = 0.00%) (Additional file 1: Fig. S16). When we sequentially removed each study from the main analysis, we observed that summary WMD changed after excluding the Yao et al. study [31] (WMD = − 4.146, 95% CI = − 6.348 to − 1.944, p < 0.001, I^2^ = 0.00%) (Additional file 1: Fig. S17). The subgroup analysis of LVESV for each imaging modality is also presented in Additional file 1: Fig. S18.

### Meta-regression analysis

Meta-regression analyses were performed between primary outcomes (hospitalization for HF, MI recurrence, mortality, and composite end-points) and age and gender. No association was found between age and long-term clinical efficacy of BM-MNC therapy (Hospitalization: *p* = 0.83, Recurrence of MI: *p* = 0.91, Mortality: *p* = 0.69, Composite end-points: *p* = 0.97) (Additional file 1: Fig. S19). Also, there was no statistically significant trend for primary end-points and gender (Hospitalization: *p* = 0.71, Recurrence of MI: *p* = 0.70, Mortality: *p* = 0.93, Composite end-points: *p* = 0.85) (Supplementary Fig. 20).

## Discussion

In this study, we have shown that transplantation of BM-MNCs after AMI improves both myocardial performance indices, such as LVEF and cardiovascular outcomes, mainly by reducing the rehospitalization rate for CHF and reinfarction rates. This treatment has not been shown to have an effect on reduction of cardiovascular death. To the best of our knowledge, this is the only meta-analysis in the field which has focused on the effect of cell therapy on MACE.

In the last two decades, many trials have been conducted to acquire a better understanding about the possible effects of stem cells transplantation on myocardial performance indices such as LVEF and scar size. However, studies focusing on clinical outcomes are rare. The BAMI trial was the first phase III trial conducted to clarify whether post-MI intracoronary transplantation of BM-MNCs would reduce all-cause mortality or not. Although the trial was designed to involve 3000 patients, it was stopped prematurely due to futility after the enrollment of 375 patients. Among them, 185 received BM-MNCs (intracoronary infusion) 2–8 days following primary percutaneous coronary intervention (PPCI), and the remaining 190 patients received optimal medical therapy as the control group. All-cause mortality after two years was 3.26% [n = 6; 95% confidence interval (CI): 1.48–7.12%] with BM-MNCs compared to 3.82% (n = 7; 95% CI: 1.84–7.84%) with optimal medical therapy [9]. The main reason behind such results was a significant reduction in post-AMI mortality. At the start of the project in 2011, the literature held that following an AMI, the mortality rate from all causes after two years would be approximately 12% among those with an LVEF ≤ 45% post-reperfusion therapy [3]. However, the researchers noticed a 3.85% mortality rate while conducting the study, reflecting the evolution of primary angioplasty procedures in those years. Our findings in this meta-analysis confirm the BAMI findings.

Post-MI heart failure appears to be a strong predictor of mortality [35]. Thus, this meta-analysis aimed to explore the potential impact of BM-MNC therapy on clinical outcomes including hospitalization for CHF. We found that transplantation of the mononuclear cells following reperfusion therapy in the setting of acute MI could significantly decrease the risk of rehospitalization due to decompensated heart failure. It should be noted that the subgroup analyses revealed that high dose (≥ 10^8^ cells) and early injection (< 11 days) of the stem cells lowered the risk of hospitalization, whereas there was no evidence of association between low dose and late injection of BM-MNCs and lower risk of hospitalization. In to the same line with our findings, BAMI investigators noticed that only five patients (2.7%, 95% CI 1.0–5.9%) who received BM-MNCs were hospitalized due to HF during the two years of follow-up compared with 15 patients (8.1%, CI 4.7–12.5%) who received optimal medical therapy (HR: 0.33, 95% CI: 0.12–0.88), representing the sole clinical benefit observed. Results from our meta-analysis and BAMI showed that taking mortality as an endpoint for stem cell therapy trials was futile, and the best clinical endpoint to assess was HF incidence.

Our results also demonstrated that injection of the mononuclear cells could cause favorable effects on LV function indices including LVEF and LVESV although there was no statistical improvement in LVEDV in the BM-MNC group when compared to the placebo group. Results regarding the effect of BM-MNC transplantation on LVEF are controversial. These controversies are mainly derived from the different protocols used in these studies. The number of the cells transplanted, route of delivery, transplantation time from AMI, age, baseline LVEF, and the method used for measuring LVEF are all affecting these outcomes. In general, most meta-analyses have shown at least a modest effect on LVEF. In a Cochrane meta-analysis, it was shown that BM-MNCs could achieve a 2.72% improvement in LVEF [36]. In a patient level data meta-analysis, it was shown that this effect might be improved in younger patients (< 55 years) and those with lower values of LVEF in the time of admission (LVEF < 40) [37]. Meanwhile, in some trials, BM-MNC therapy failed to improve the LV function including LVEF, regional LV function, and wall motion in the infarct zone [24, 25]. Although the effect of BM-MNC infusion on LVEF seems to be small, it should be noted that other treatment modalities such as beta blocker therapy or direct revascularization also have a relatively small influence on LVEF improvement [37]. Thus, a more important question would be the long-term effects of this treatment on clinical outcomes and that is where our study has focused on.

## Limitations

There were some limitations to our analysis that should be taken into account. As with any meta-analysis, limitations to the method include heterogeneity across trials. In particular, there are differences in terms of treatment characteristics including the cell dosage used, cell isolation protocols, storage methods, and image modalities. Furthermore, the primary outcome of many studies was LVEF, and these studies were not designed specifically to monitor major cardiovascular events.

## Conclusion

In conclusion, injection of BM-MNC in patients with acute MI may contribute to lower risk of long-term hospitalization for CHF and recurrence of MI, especially when administered in high doses and shortly after the reperfusion therapy. However, despite a lower numerical rate of cardiovascular mortality this treatment does not reach statistical significance. BM-MNC therapy could also result in significant improvements in LV function indices including LVEF and LVESV in the follow up period compared to the patients receiving standard therapy. The results of this meta-analysis showed that transplantation of BM-MNCs can have a substantial effect on clinical and paraclinical outcomes.

## Supplementary Information

**Additional file 1:** Supplementary material.

## Data Availability

The data underlying this article will be shared on reasonable request to the corresponding author.

## Abbreviations

MI: Myocardial infarction
AMI: Acute myocardial infarction
HF: Heart failure
CHF: Congestive heart failure
BM-MNC: Bone marrow mononuclear cell
LVEF: Left ventricular ejection fraction
LVEDV: Left ventricular end-diastolic volume
LVESV: Left ventricular end-systolic volume
RR: Risk ratio
CI: Confidence interval
WMD: Weighted mean difference
MACE: Major adverse cardiovascular events
CMR: Cardiac magnetic resonance
SPECT: Single-photon emission computed tomography
PPCI: Primary percutaneous coronary intervention
