# Supplementary material for: Major cardiovascular events after bone marrow mononuclear cell transplantation following acute myocardial infarction: an updated post-BAMI meta-analysis of randomized controlled trials
Source: BMC Cardiovasc Disord. 2022 Jun 9;22:259. doi: 10.1186/s12872-022-02701-x (PMC9185901; doi:10.1186/s12872-022-02701-x)
Supplement: Supplementary file 1 — Additional file 1: Supplementary material. [file 12872_2022_2701_MOESM1_ESM.docx]

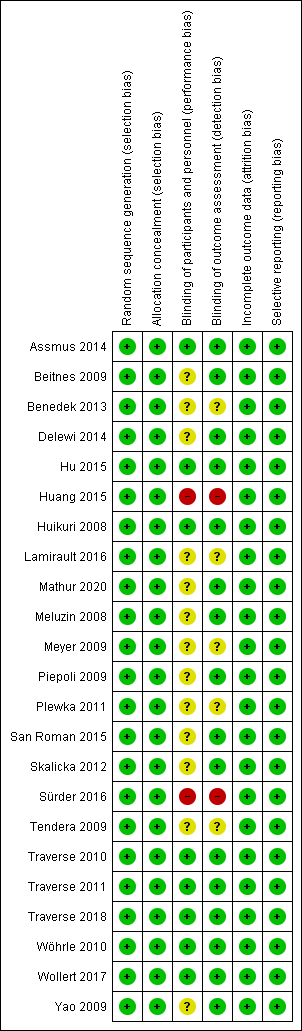


Figure 1: Risk of bias assessment of the enrolled studies


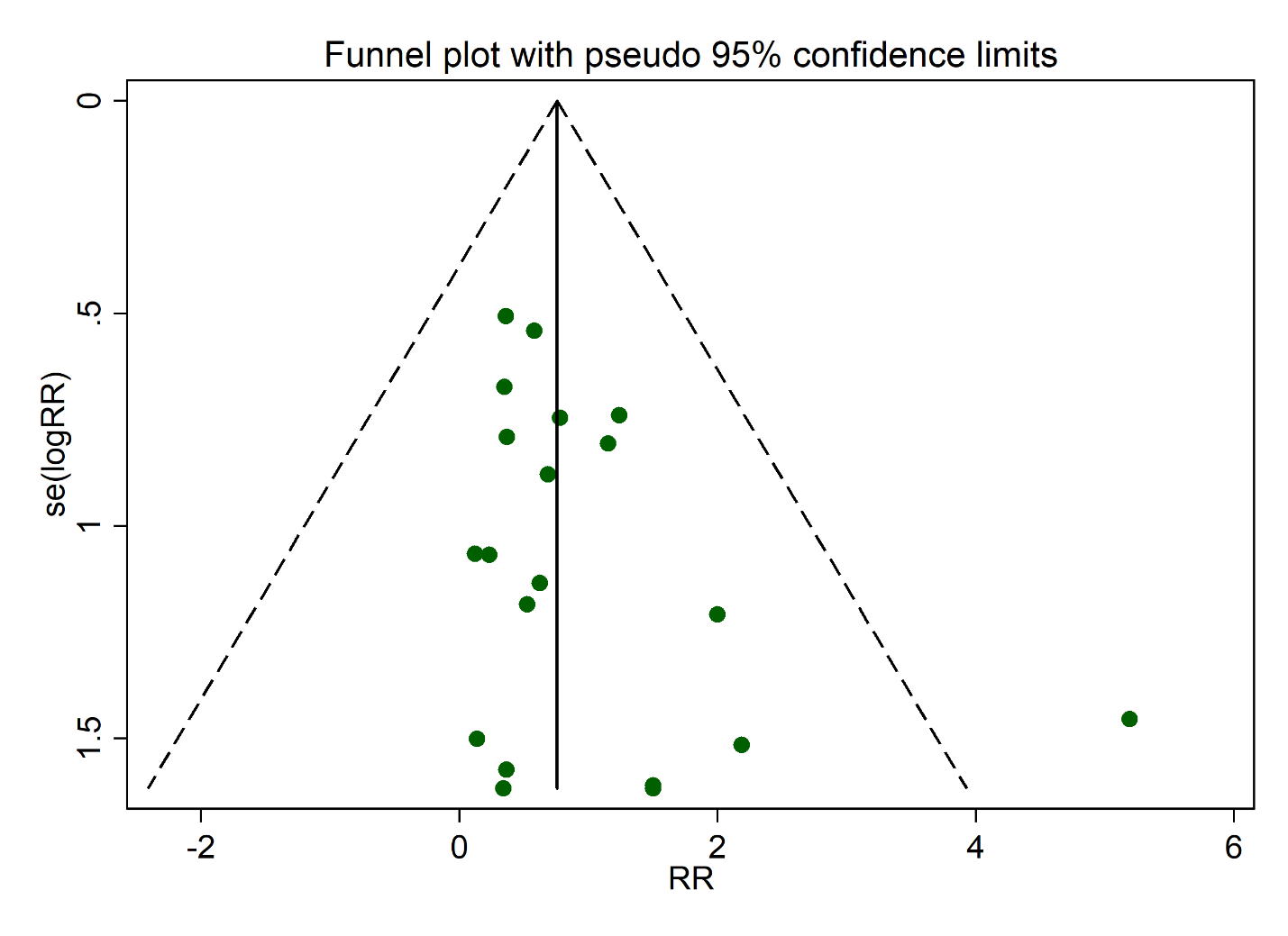


Figure 2: Funnel plot of hospitalization for CHF


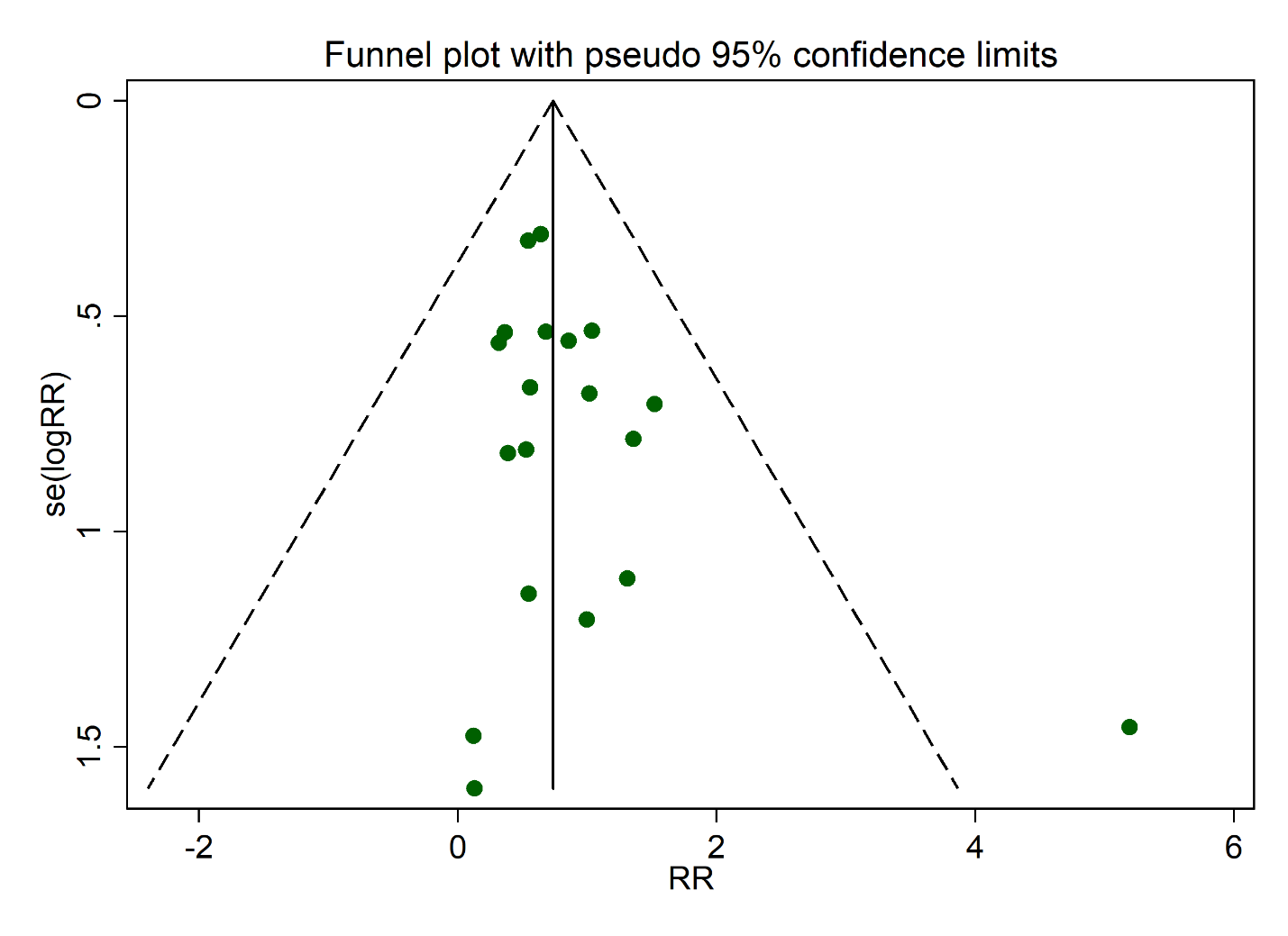


Figure 3: Funnel plot of composite endpoints


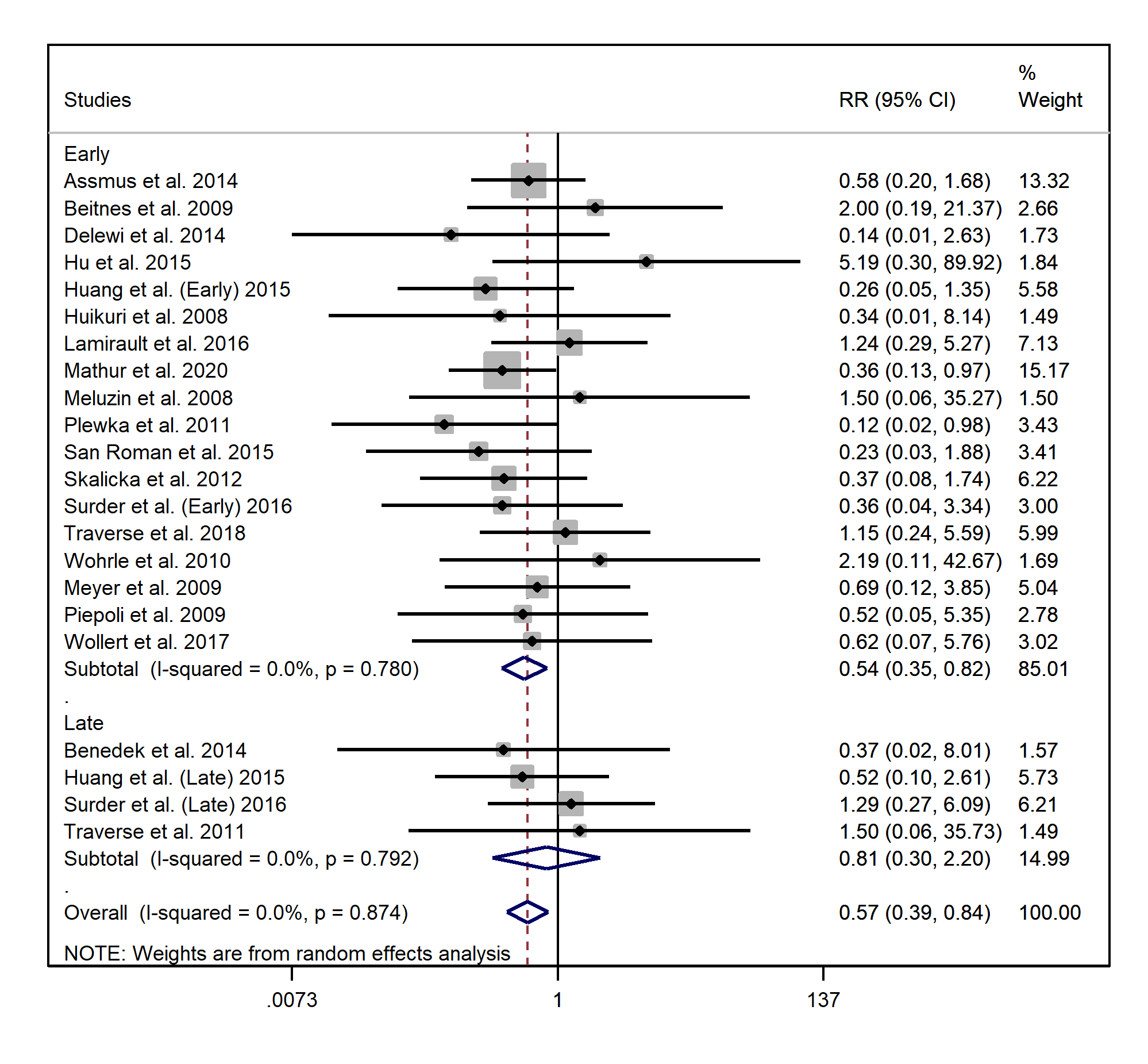


Figure 4: Relative risk of hospitalization for CHF in early and late injection group


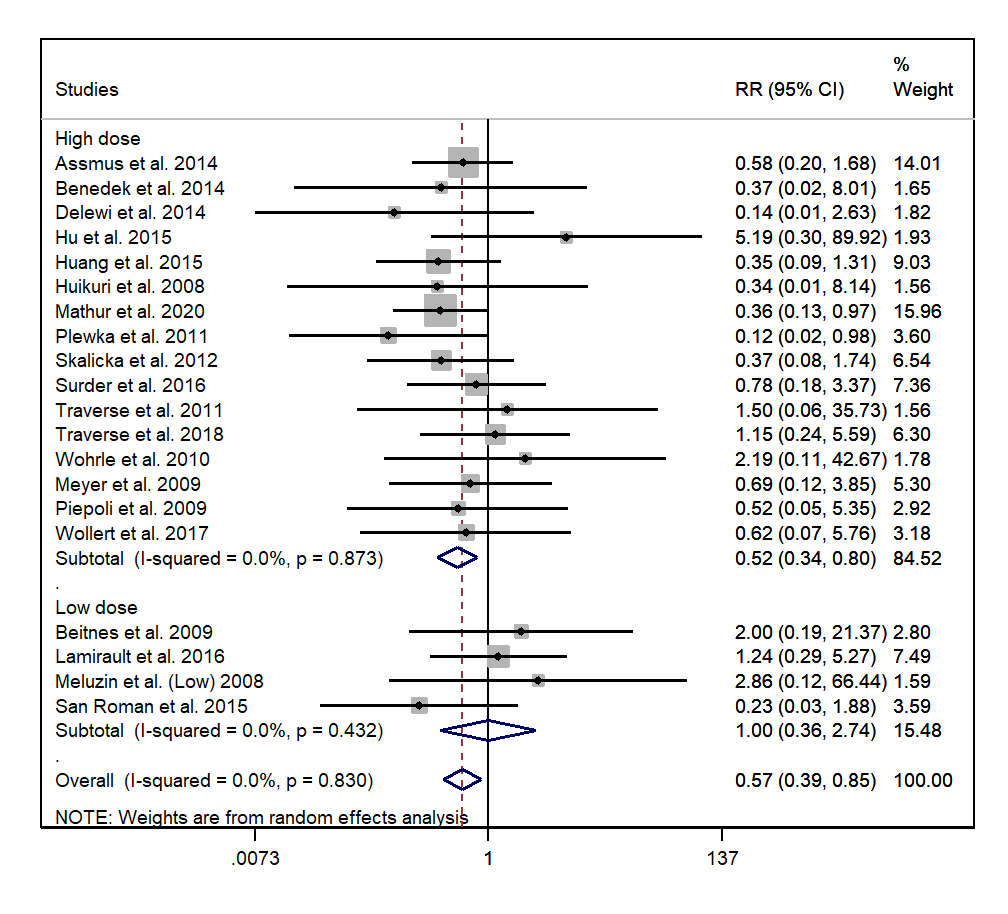


Figure 5: Relative risk of hospitalization for CHF in low and high dose group


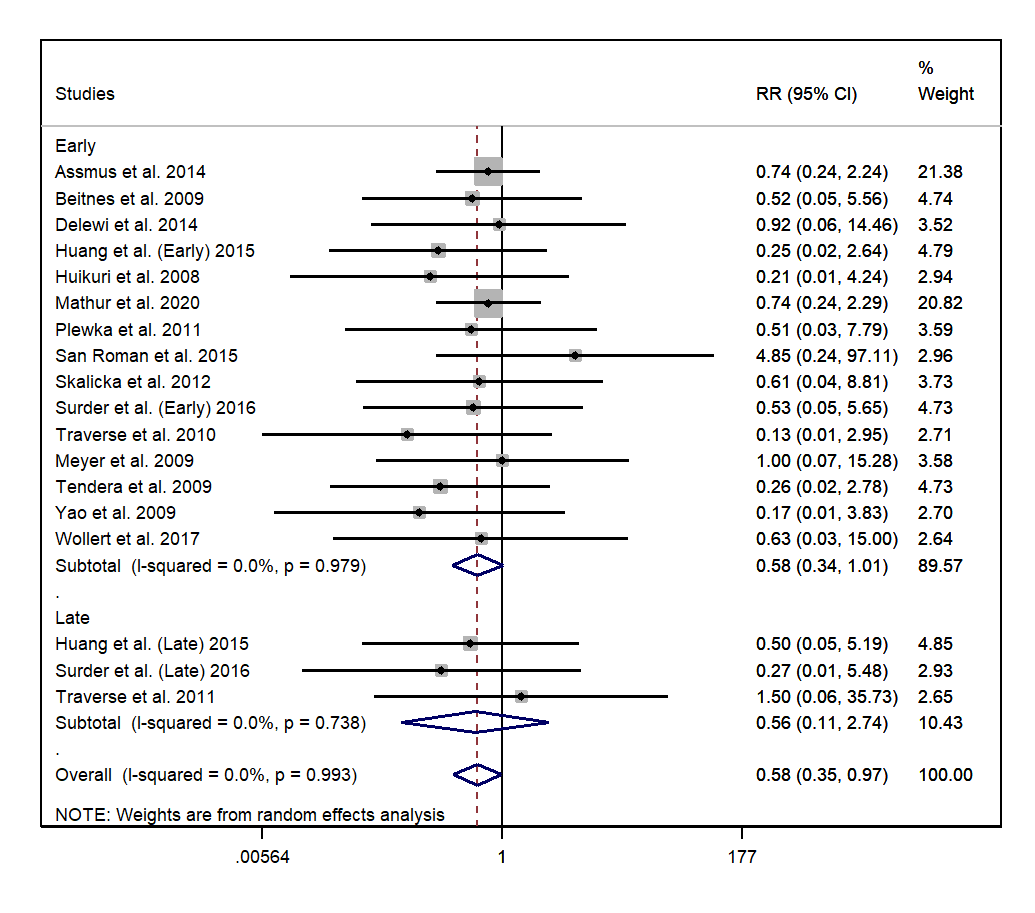


Figure 6: Relative risk of myocardial reinfarction in early and late injection


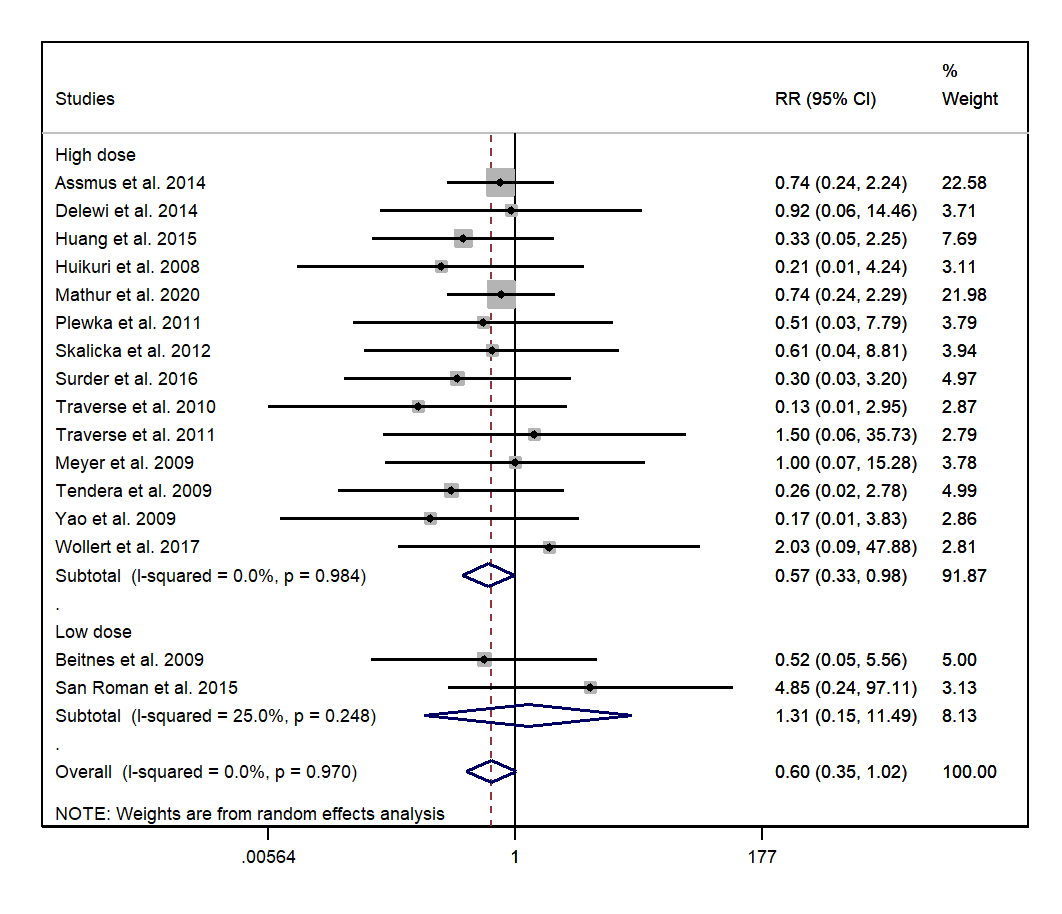


Figure 7: Relative risk of myocardial reinfarction in high and low dose of injection


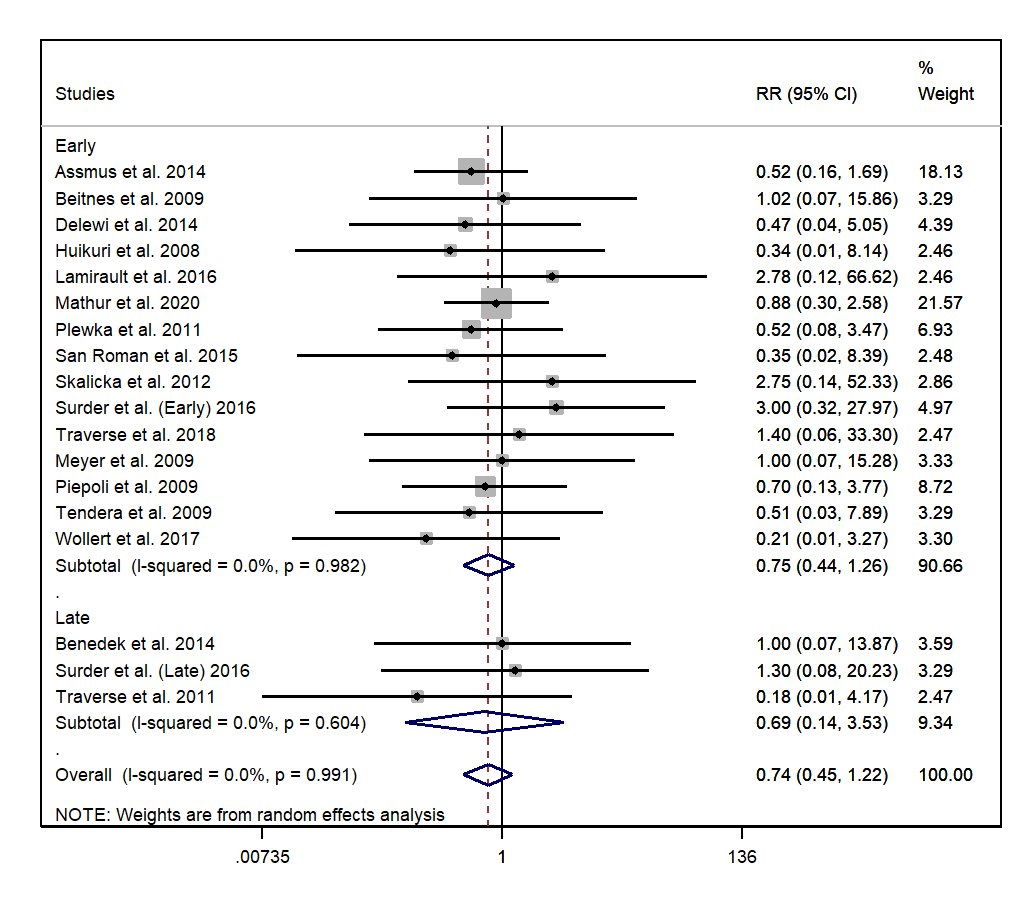


Figure 8: Relative risk of cardiac-related mortality in early and late injection


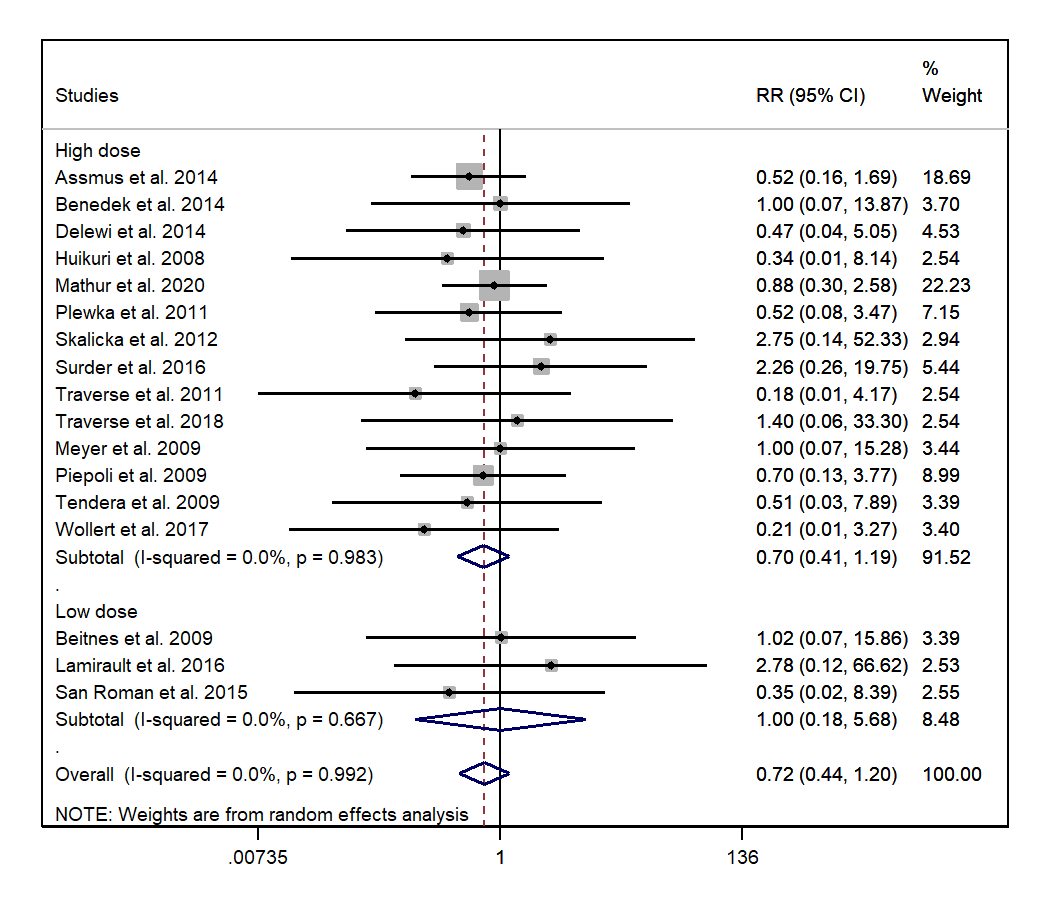


Figure 9: Relative risk of cardiac-related mortality in high and low dose injection


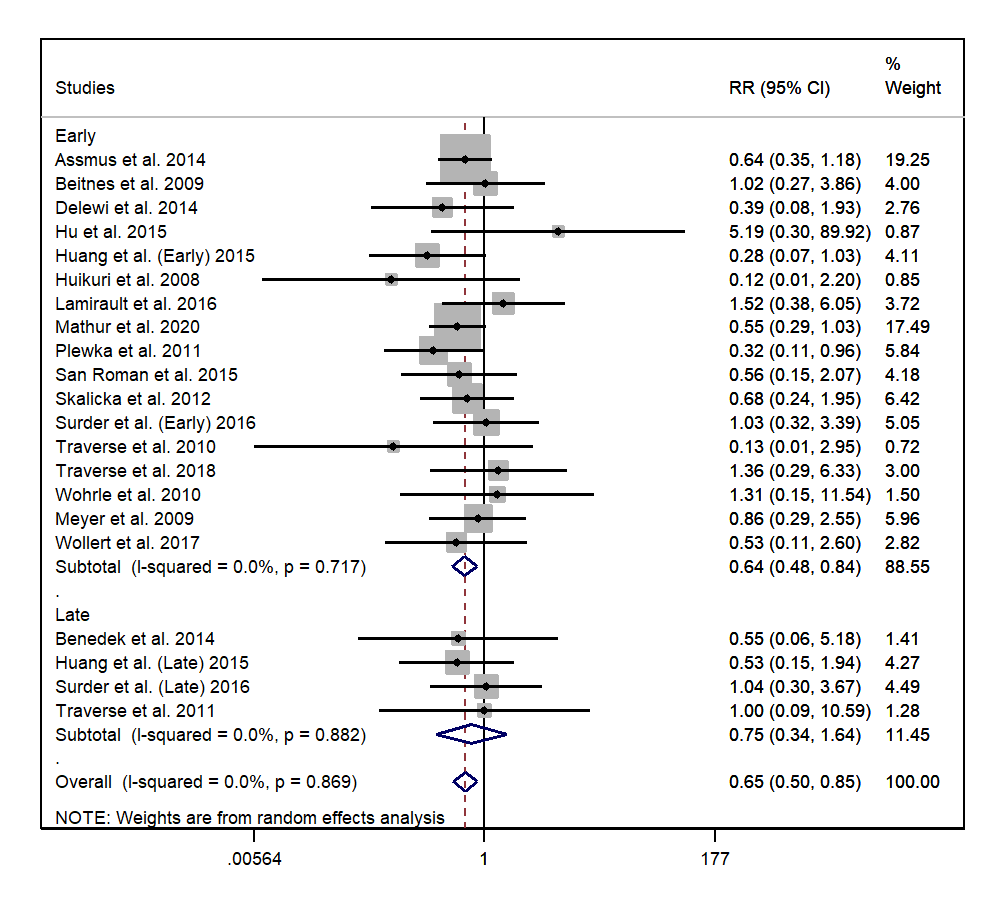


Figure 10: Relative risk of composite endpoints in early and late group


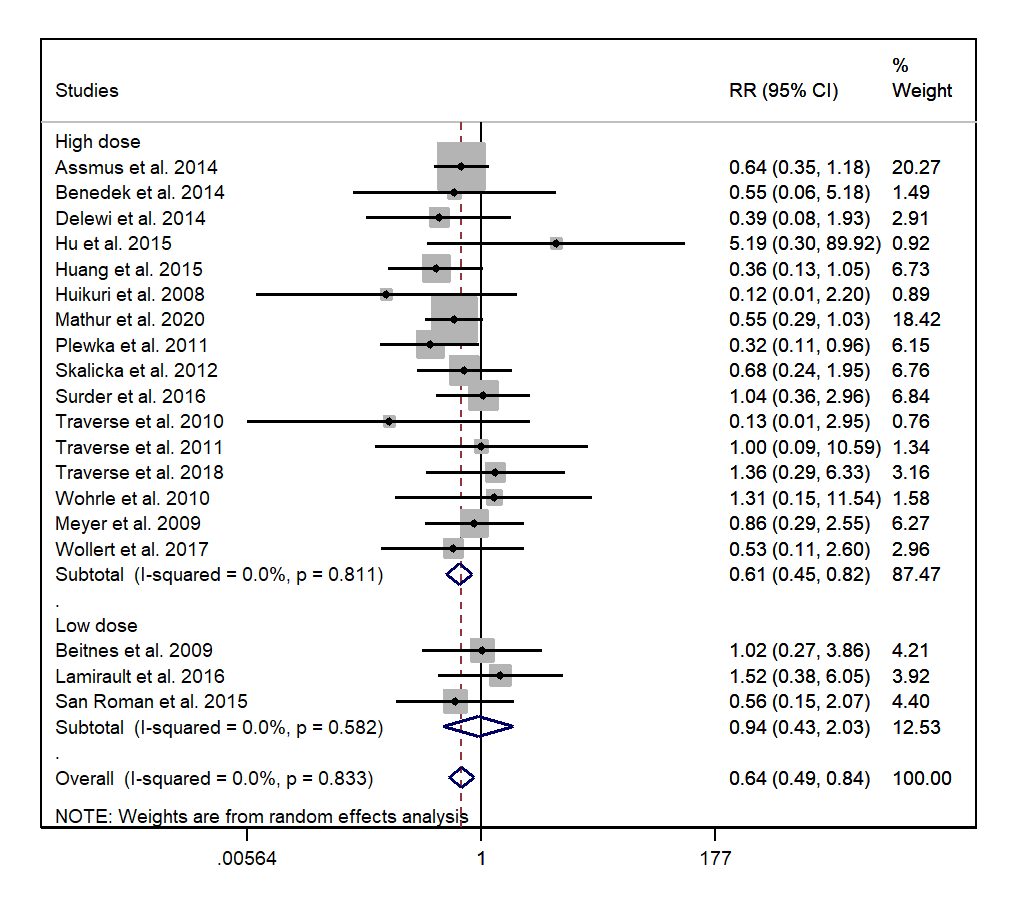


Figure 11: Relative risk of composite endpoint in high and low dose group


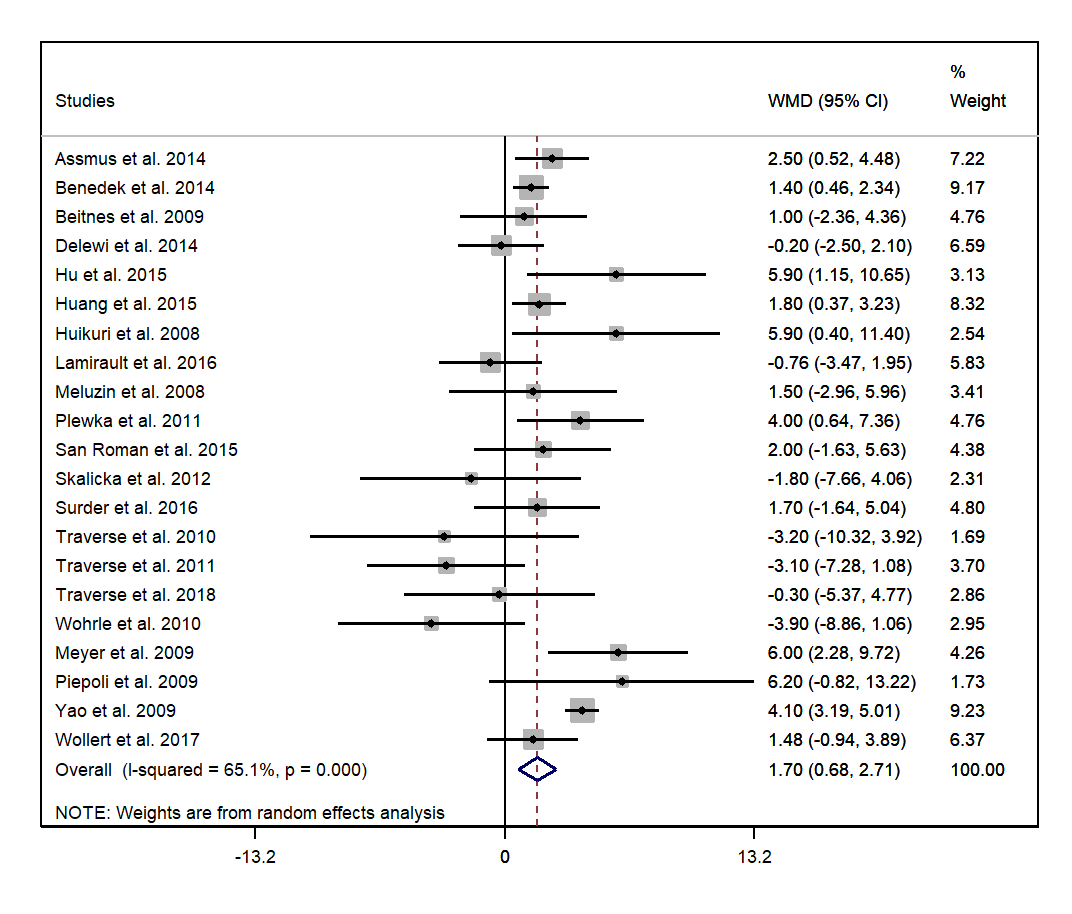


Figure 12: Forest plot of LVEF improvement compared between control and intervention group


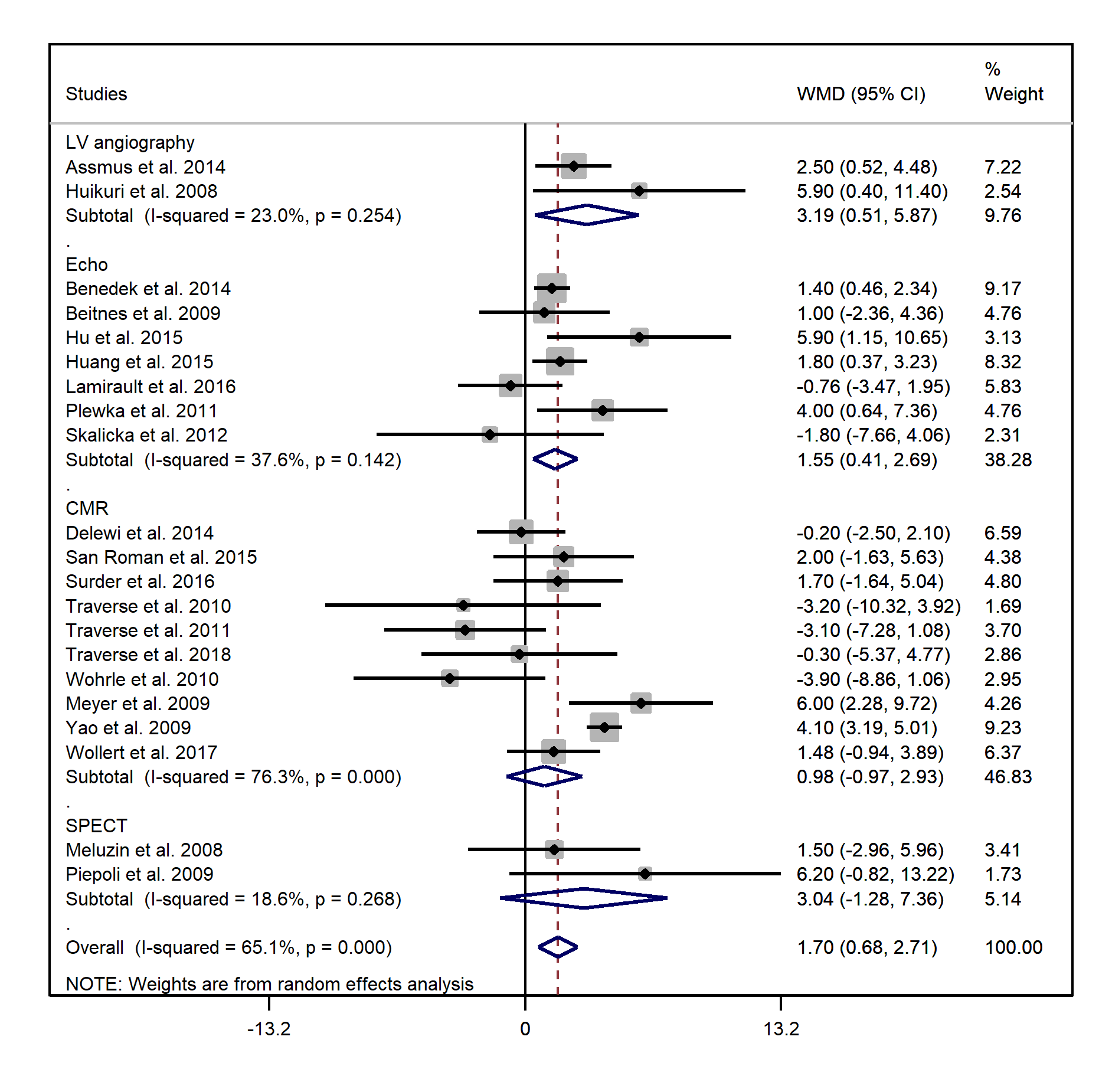


Figure 13: Forrest plot of subgroup analysis of LVEF improvement measured by each modality


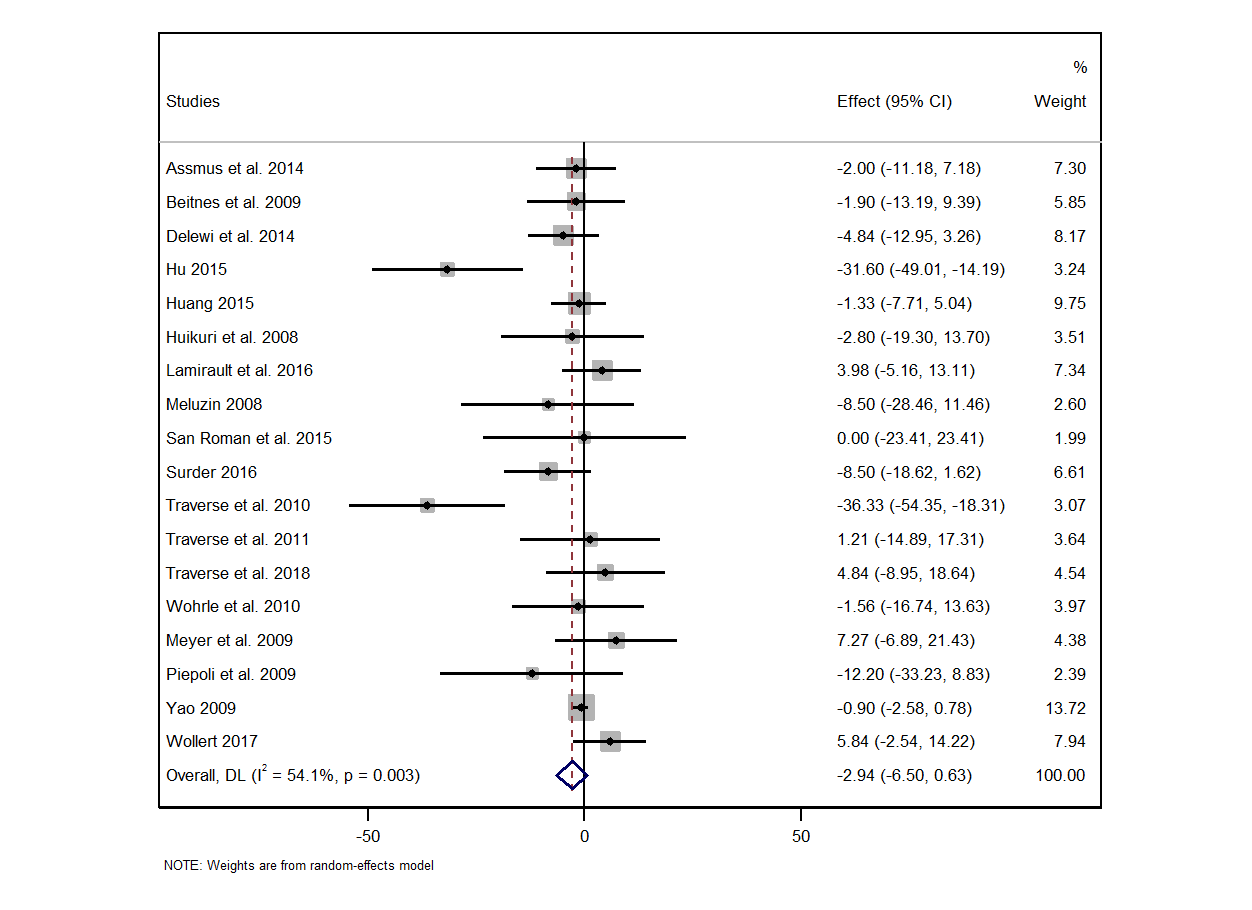


Figure 14: Forest plot of changes in LVEDV values for all modalities


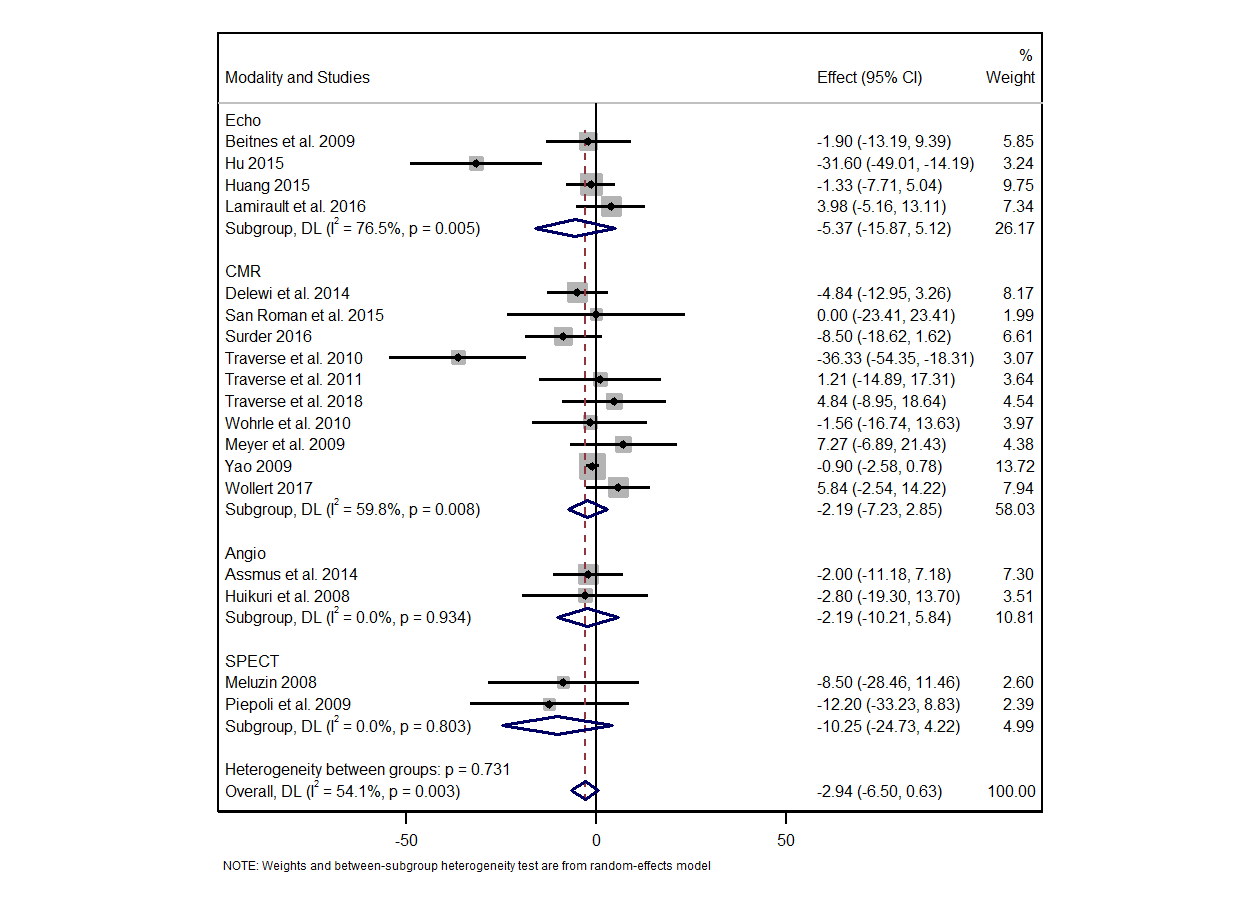


Figure 15: Forest plot of subgroup analysis of changes in LVEDV based on each modality


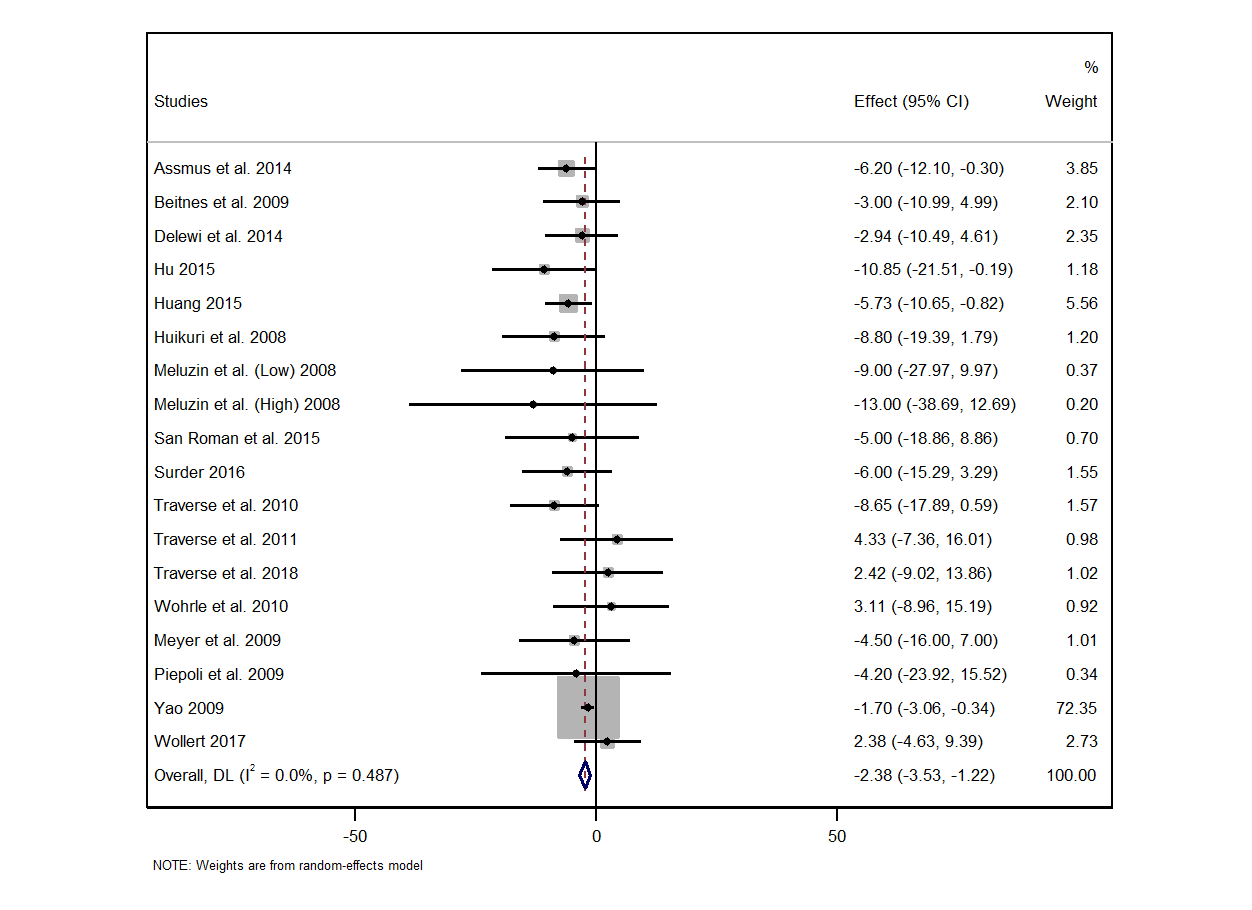


Figure 16: Forest plot of changes in LVESV before excluding the study by Yao et al.


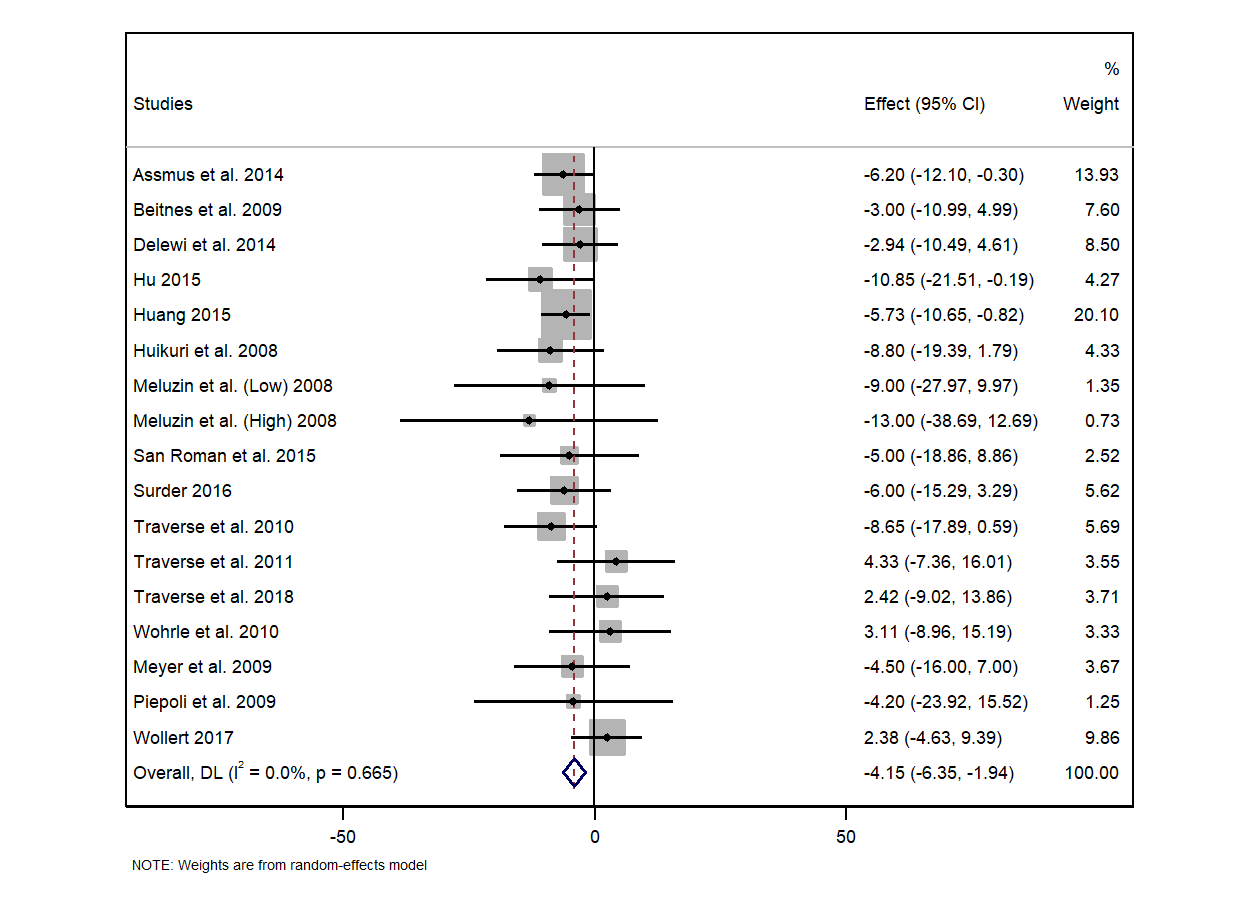


Figure 17: Forest plot of changes in LVESV after excluding the study by Yao et al.


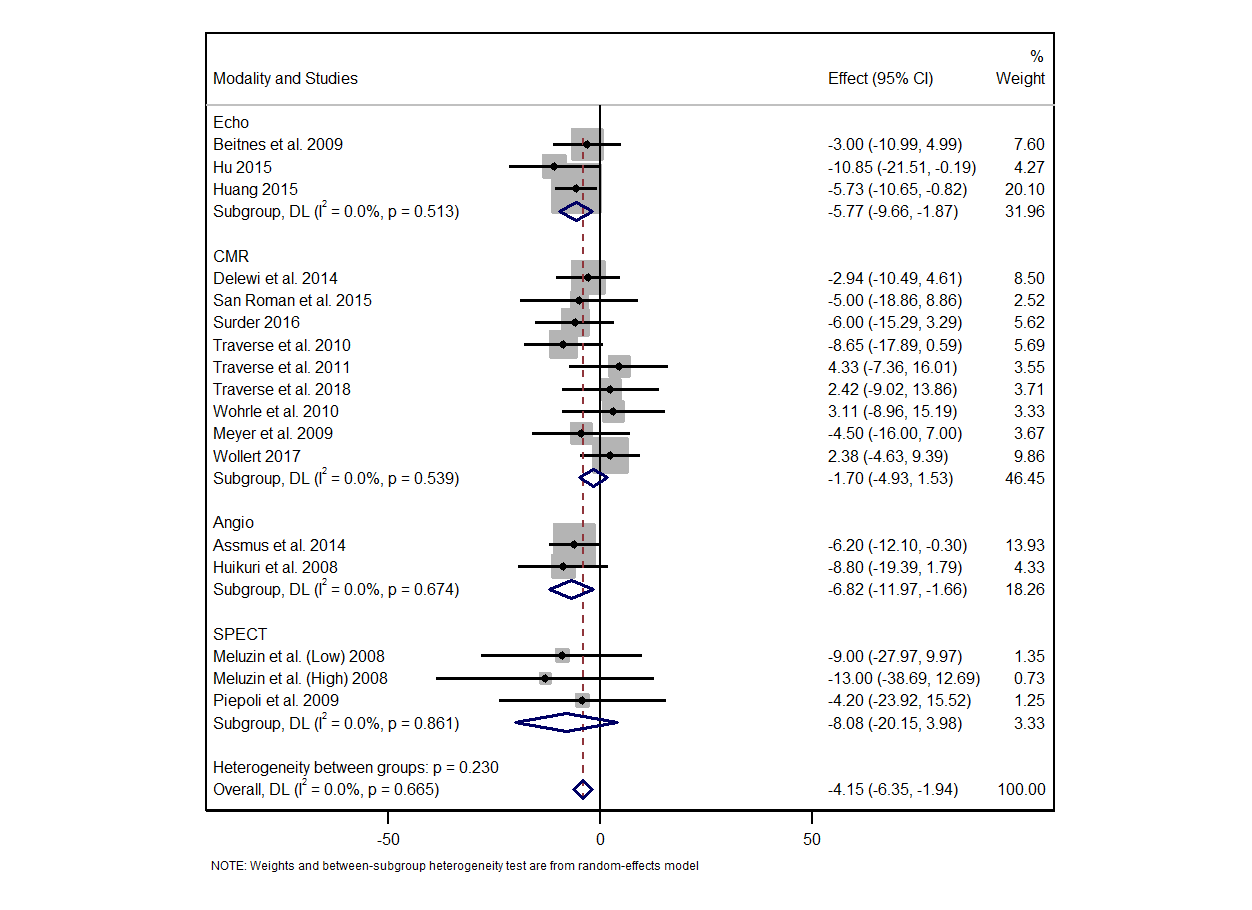


Figure 18: Forest plot of subgroup analysis changes in LVESV


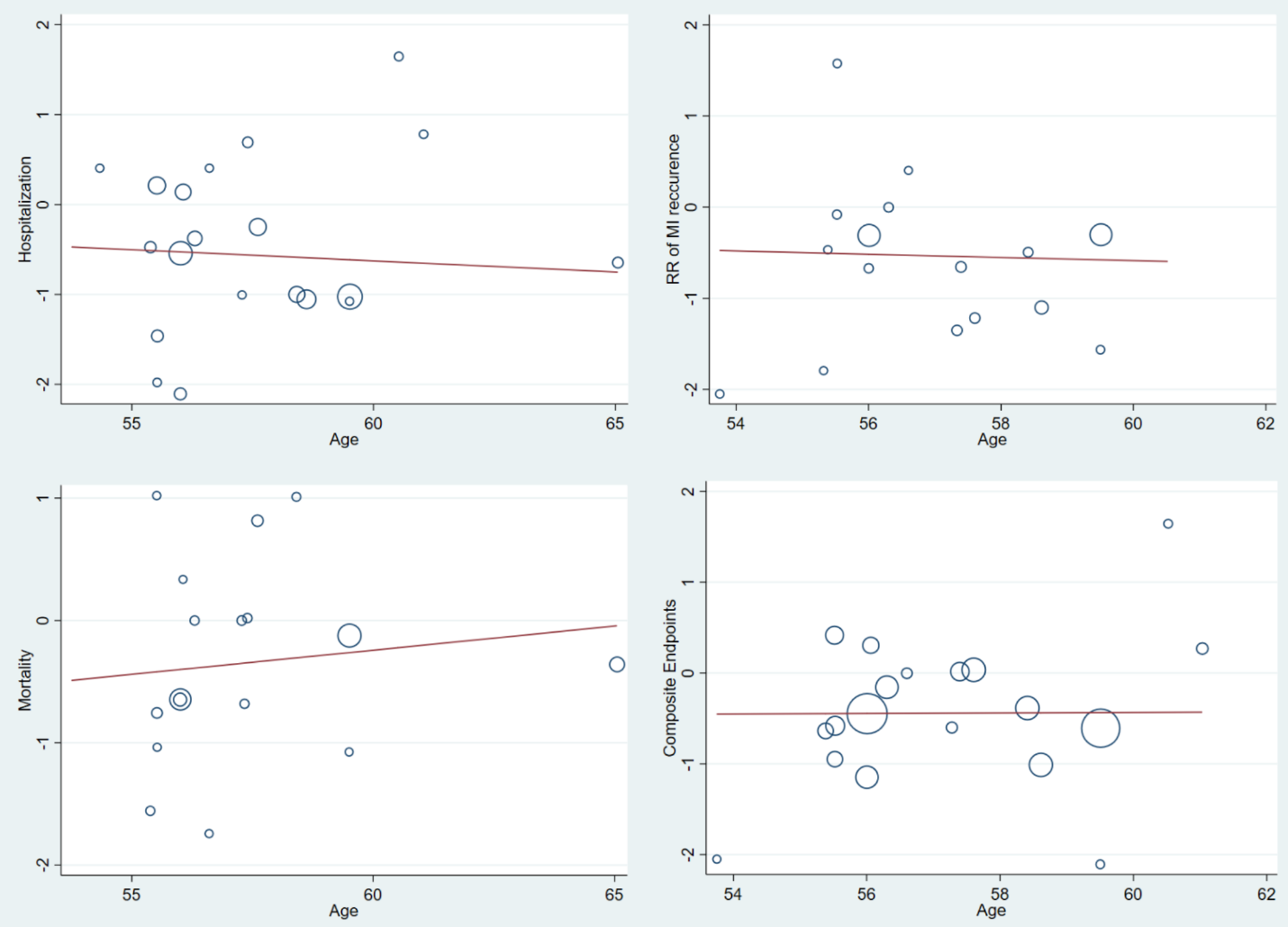


Figure 19: Meta-regression for primary end-points based on age


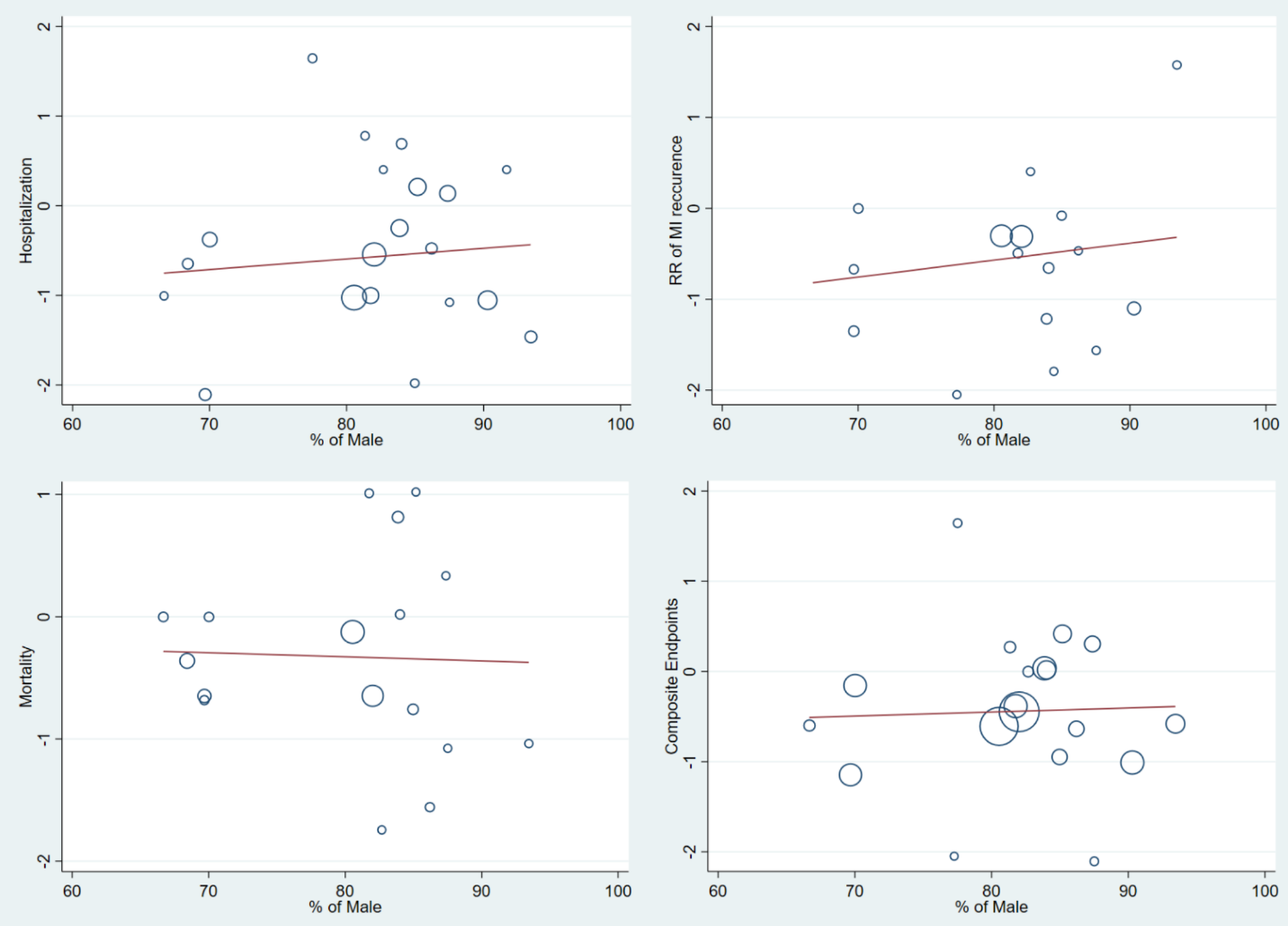


Figure 20: Meta-regression for primary end-points based on percentage of males
